# Inhibition of Membrane-Associated Catalase, Extracellular ROS/RNS Signaling and Aquaporin/H_2_O_2_-Mediated Intracellular Glutathione Depletion Cooperate during Apoptosis Induction in the Human Gastric Carcinoma Cell Line MKN-45

**DOI:** 10.3390/antiox10101585

**Published:** 2021-10-09

**Authors:** Georg Bauer

**Affiliations:** Medical Center, Faculty of Medicine, Institute of Virology, University of Freiburg, D-79104 Freiburg, Germany; georg.bauer@uniklinik-freiburg.de

**Keywords:** hydrogen peroxide, aquaporin, catalase, tumor cell, apoptosis-inducing signaling, glutathione, xC transporter, apoptosis

## Abstract

The human gastric carcinoma cell line MKN-45 is a prototype of bona fide tumor cells, as it is protected from the NADPH oxidase-1 (NOX-1)-driven HOCl- and nitric oxide (NO)/peroxynitrite apoptosis-inducing signaling pathways by a membrane-associated catalase. The use of inhibitors/scavengers shows that inhibition of membrane-associated catalase is sufficient for the activation of NO/peroxynitrite or HOCl signaling. However, this signaling is not sufficient for apoptosis induction, as intracellular glutathione peroxidase/glutathione counteracts these signaling effects. Therefore, intrusion of extracellular tumor cell-derived H_2_O_2_ through aquaporins is required for the full apoptosis-inducing effect of extracellular reactive oxygen/nitrogen species. This secondary step in apoptosis induction can be prevented by inhibition of aquaporins, inhibition of NOX1 and decomposition of H_2_O_2_. Pretreatment with inhibitors of glutathione synthase or the cysteine-glutamine antiporter (xC transporter) abrogate the requirement for aquaporin/H_2_O_2_-mediated glutathione depletion, thus demonstrating that intracellular glutathione is the target of intruding H_2_O_2_. These data allow definition of mechanistic interactions between ROS/RNS signaling after inhibition of membrane-associated catalase, the sensitizing effects of aquaporins/H_2_O_2_ and the counteraction of the xC transporter/glutathione synthase system. Knowledge of these mechanistic interactions is required for the understanding of selective apoptosis induction in tumor cells through reestablishment of apoptosis-inducing ROS/RNS signaling.

## 1. Introduction

The demonstration of a steady state level of compound I of catalase in hemoglobin-free perfused rat liver by Sies and Chance [1] was the first proof for the generation of H_2_O_2_ under physiological conditions. This finding opened the path for the elucidation of numerous physiological and pathophysiological roles of H_2_O_2_, as well as its control by enzymes such as catalase, peroxidase and others [2,3,4]. An involvement of H_2_O_2_ in apoptosis induction was first recognized by Parchent and Pierce et al. [5,6]. Since then, a complex network of direct and indirect H_2_O_2_ actions on the control of proliferation, gene expression, angiogenesis, immune mechanisms, function of phagocytes and many other biological processes has been elucidated, as reviewed by [3,4,7,8]. Sies [8] pointed out that the concentration range of H_2_O_2_ determined whether it induced signaling effects, adaptive responses or damage.

One classical mechanism for H_2_O_2_-mediated damaging effects is based on Fenton chemistry, a process in which an electron derived from ferrous iron causes decomposition of H_2_O_2_ into hydroxyl anions and highly reactive hydroxyl radicals [9]. As this reaction lacks site-specificity, sufficiently high concentrations of H_2_O_2_ are needed to cause damage through this mechanism. Myeloperoxidase is the classical peroxidase that uses H_2_O_2_ as the substrate for the generation of HOCl [10]. Meanwhile, peroxidasin and dual oxidase (DUOX) have also been shown to generate HOCl after initial interaction with H_2_O_2_ [11]. The reaction product HOCl is essential for the antimicrobial function of granulocytes, mediates apoptosis induction selectively in malignant cells, is involved in inflammatory reactions and also in the enhancement of immune recognition [10,11,12]. Compared to the reaction of H_2_O_2_, the Fenton reaction of HOCl is about 1000-fold more efficient [13]. In addition, the potential of HOCl to generate hydroxyl radicals after its reaction with superoxide anions [14] is important for the specificity of the biological effects of HOCl [11,15,16]. This is due to the relatively short free diffusion path length of superoxide anions and hydroxyl radicals [17,18]. Therefore, the interaction between superoxide anions and HOCl opens the chance for direct hydroxyl radical generation specifically to the site of superoxide anion generation. This principle is of explicit importance for apoptosis induction in malignant cells, which specifically express active NOX1 on their membrane. The resultant superoxide anions in the vicinity of the membrane of malignant cells enable site-specific generation of apoptosis-inducing hydroxyl radicals after superoxide/HOCl interaction [11,15].

H_2_O_2_ plays several partially dualistic roles in malignant cells. Dismutation of extracellular superoxide anions generated by membrane-associated NOX1 of oncogenically transformed cells leads to H_2_O_2_ that enters the cells and controls their proliferation, as well as the maintenance of their transformed state [19,20,21,22] (reviewed in [23,24]). This stimulating effect of H_2_O_2_ for the malignant cells is counteracted by the involvement of cell-derived H_2_O_2_ for the generation of HOCl that selectively endangers the survival of malignant cells through the HOCl signaling pathway [11]. The balance between these two opposite effects of H_2_O_2_ seems to determine the success of tumor progression. Therefore, the classical findings by Deichman’s group on the acquisition of a “H_2_O_2_-catabolizing phenotype” during tumor progression is the central part of a scheme in which the tumor cells, i.e., late stages of tumor progression, are selected for resistance towards apoptosis induction by exogenous ROS [25,26,27,28,29]. The essential resistance principle of tumor cells, according to Deichman’s concept, has been determined as membrane-associated catalase [11,30,31,32]. Membrane-associated catalase is missing on nonmalignant cells [30,31]. Membrane-associated catalase decomposes extracellular H_2_O_2_ and thus prevents DUOX-mediated HOCl synthesis [30,31] and subsequent HOCl signaling-mediated the selective apoptosis induction of malignant cells. It is obvious that this tight control of the concentration of H_2_O_2_ derived from NOX1-dependent superoxide anions protects the tumor cells, but at the same time poses a serious problem for their need to control their proliferation by extracellular H_2_O_2_.

Aquaporins facilitate the exchange of water through membranes, but some members of the aquaporin family (AQP 1, 3, 55, 8, 9) are also efficient transporters of H_2_O_2_ that enable the influx of exogenous H_2_O_2_ into cells [33,34]. This results in several subsequent H_2_O_2-_dependent signaling processes, with stimulation of proliferation by H_2_O_2_ as a very prominent one. An increased expression of aquaporins by tumor cells [35] thus may reflect the necessary response of the cells to cope with limited supply with exogenous H_2_O_2_, which is caused by H_2_O_2_ decomposition by membrane-associated catalase. Therefore, tumor cells with increased numbers of aquaporins may have a better chance for selection during tumor progression, as the intrusion of sufficient H_2_O_2_ for proliferation stimulation is ensured despite the high local concentration of catalase immobilized on the cell membrane.

A dominant function of aquaporins during apoptosis induction in tumor cells after treatment with cold atmospheric plasma, or plasma-activated medium, was confirmed in recent studies [36,37,38,39,40,41]. Thereby, a kinetic analysis showed that aquaporins seemed to play a role in the time window between inactivation of protective catalase by plasma and the onset of effective intercellular apoptosis-inducing signaling [38,40].

The intracellular level of glutathione is determined by the rates of its synthesis and consumption. Therefore, the activity of the xC transporter that controls the influx of cystine is of rate-determining importance [42,43]. Cysteine, derived from cystine is required for the synthesis of glutathione. The xC reporter has come into the focus of oncology recently as many tumor cells show overexpression of the xC transporter and inhibition of the transporter causes synergistic effects with other treatment regimes. Bekeschus et al. reported on the controlling function of the xC transporter for apoptosis induction by CAP in several tumor cell lines [44]. Overexpression of the xC transporter caused protection against the apoptosis-inducing effects of CAP.

This study is based on the working hypothesis that the established reactivation of extracellular NO/peroxynitrite and HOCl signaling of tumor cells after reactivation of membrane-associated catalase is not necessarily sufficient for the induction of apoptosis, as it may be counteracted by the interaction of intracellular glutathione and glutathione peroxidase, which counteract the effects of the ROS/RNS signaling on the membrane. Triggered by the findings by Yan and Keidar [36,37], and based on previous own data on the potential role of aquaporins for sensitizing tumor cells for extracellular ROS/RNS signaling [38], our working hypothesis assumed that the influx of extracellular, tumor-cell derived H_2_O_2_ through aquaporins may interfere with glutathione/glutathione peroxidase-mediated protection and thus facilitate apoptosis induction by extracellular ROS/RNS signaling.

This study utilized MKN-45 human gastric carcinoma cells, as this cell line has been characterized as a prototype of bona fide tumor cells that shows the ROS/RNS-relevant features of tumor cells according to the concept of multistep oncogenesis following the concepts of Deichman and colleagues [25,26,27,28,29], Irani et al. and others [19,20,21,22], and Bauer et al. [23,24]. MKN-45 cells express membrane-associated NADPH oxidase-1 (NOX1) and thereby generate extracellular superoxide anions—a hallmark and requirement of the malignant state [19,20,21,22,23]. Extracellular superoxide anions drive the NO/peroxynitrite and the HOCl signaling pathway, which causes selective apoptosis induction in malignant cells [11,15,30,45,46]. MKN-45 cells prevent NO/peroxynitrite and HOCl signaling through the expression of membrane-associated catalase that interferes with both apoptosis-inducing pathways [23,24,30,31]. Protection against ROS/RNS signaling seems to be a general and specific feature of bona fide tumor cells [23,24,30,31]. Therefore, induction of RNS/ROS-dependent apoptosis inducing signaling requires the inhibition or inactivation of membrane-associated catalase. This principle has been demonstrated in the case of MKN-45 cells for (a) direct catalase inhibitors, such as 3-AT, salicylic acid, neutralizing antibodies, antibody Fab fragments, single domain antibodies [30,31,47,48], (b) inhibition of transglutaminase, that prevents the binding of catalase to the membrane [49], (c) addition of modulators of the NO metabolism such as arginine, arginase inhibitors, inhibitors of NO dioxygenase, interferon gamma [47,50], (d) direct application of catalase-inactivating singlet oxygen [51], (e) singlet oxygen generated by physical plasma or plasma-activated medium [38,39,40,41] and (f) by siRNA-mediated knockdown of catalase [49]. Apoptosis induction in MKN-45 cells through ROS/RNS signaling after catalase inhibition/inactivation has been dissected through the use of scavengers/inhibitors, reconstitution experiments based on the addition of defined signaling components and siRNA-based analysis [30,49,52]. Based on these established data, and as MKN-45 cells allow a gradual shift between NO/peroxynitrite and HOCl signaling, and therefore allow to study the effects of or on both signaling pathways, this cell line was found to be optimal in studying additional signaling features. These were the counteraction of intracellular glutathione/glutathione peroxidase and the requirement to abrogate this counteraction through aquaporin-mediated H_2_O_2_ influx. It seems appropriate that additional branches of signaling chemistry that were elucidated in this way might later be tested for relevance in other tumor cell systems that are also defined by the classical NOX1/catalase connection.

## 2. Materials and Methods

### 2.1. Materials

The NOX1 inhibitor 4-(2-Aminoethyl)benzenesulfonyl fluoride (AEBSF), the aquaporin inhibitor AgNO_3_, the catalase inhibitor 3-aminotriazole (3-AT), the inhibitor of glutathione synthesis buthionine sulfoximine (BSO), catalase from bovine liver, cyanidin, the hydroxyl radical scavenger mannitol, the fast decaying NO donor diethylamine NONOate (DEA NONOate), glucose oxidase (GOX), the singlet oxygen scavenger histidine, hydrogen peroxide, the hydroxyl radical scavenger mannitol, myeloperoxidase, the NOS inhibitor N-omega-nitro-L-arginine methyl ester hydrochloride (L-NAME), potassium nitrite, the HOCl scavenger taurine, taxol (paclitaxel), Mn-SOD from E. coli, neutralizing monoclonal antibodies against catalase (clone CAT-505, mouse, IgG1), monoclonal antibodies directed against laminin, and sulfasalazine, were obtained from Sigma-Aldrich (Schnelldorf, Germany). The peroxidase inhibitor 4-Aminobenzoyl hydrazide (ABH) was obtained from Acros Organics (Geel, Belgium). Inhibitors for caspase-3 (Z-DEVD-FMK), and caspase-9 (Z-LEHD-FMK) were obtained from R&D Systems (Wiesbaden-Nordenstadt, Germany). Peroxynitrite and the peroxynitrite decomposition catalysts 5-, 10-, 15-, 20-Tetrakis(4-sulfonatophenyl)porphyrinato iron(III) chloride (FeTPPS), were obtained from Calbiochem (Merck Biosciences GmbH, Schwalbach/Ts, Germany). The catalase mimetic EUK-134 [chloro([2,2′-[1,2-ethanediylbis[(nitrilo-κN)methylidyne]]bis [6-methoxyphenolato-κO]]]-manganese was a product of Cayman (Ann Arbor, MI, USA) and was obtained from Biomol (Hamburg, Germany). Photofrin (a product of Axcan, Quebec, Canada) was obtained from Meduna Arzneimittel GmbH (Aschaffenburg, Germany). N^ω^-Hyroxy-nor-L-arginine.acetate (NOR-NOHA) was obtained from Axxora/Enzo Life Sciences, Lörrach, Germany. Detailed information on inhibitors has been previously published [53,54,55,56]. The site of action of inhibitors and scavengers has been presented in detail in the Supplementary Materials of references [55,56]. Please find further information on the site of action of inhibitors/scavengers and on data confirming their specific action under Supplementary Materials.

### 2.2. Cells and Cell Culture

The human gastric adenocarcinoma cell line MKN-45 (ACC 409) (established from the poorly differentiated adenocarcinoma of the stomach (medullary type) of a 62-year-old woman), was purchased from DSMZ, Braunschweig, Germany.

MKN-45 cells were cultured in RPMI 1640 medium containing 10% fetal bovine serum (FBS). MKN-45 cells show active membrane-associated NOX-1, resulting in the generation of extracellular superoxide anions and are protected against intercellular ROS/RNS-dependent apoptosis-inducing signaling through expression of membrane-associated catalase [30,31]. MKN-45 cells have been found to represent an adequate model cell line to study apoptosis-inducing NO/peroxynitrite and HOCl signaling after inhibition of their membrane-associated catalase [30].

Fetal bovine serum (Biochrom, Berlin, Germany) was heated for 30 min at 56 °C prior to use. Medium was supplemented with penicillin (40 U/mL), streptomycin (50 µg/mL), neomycin (10 µg/mL), moronal (10 U/mL) and glutamine (280 µg/mL). Care was taken to avoid cell densities below 300,000/mL and above 10^6^/mL.

### 2.3. Quantification of Apoptosis Induction

Autocrine apoptosis induction by intercellular ROS/RNS signaling. Tumor cells in complete medium were seeded in 96-well tissue culture clusters at a density of 12,500 cells/100 µL. Reactivation of intercellular apoptosis-inducing ROS/RNS signaling required inactivation of membrane-associated catalase of tumor cells. Assays received the indicated concentrations of the catalase inhibitor 3-AT or other compounds, as indicated in the figures. In inhibitor studies, inhibitors were applied 10 min before 3-AT, or as indicated in the respective legends. In all experiments, assays were performed in duplicate. After the indicated time of incubation at 37 °C and 5% CO_2,_ that allowed intercellular ROS-mediated apoptosis induction, the percentage of apoptotic cells was determined by inverted phase contrast microscopy based on classical criteria for apoptosis as described below.

Determination of the percentage of apoptotic cells. After the indicated time of incubation at 37 °C and 5% CO_2_, the percentage of apoptotic cells was determined by inverted phase contrast microscopy based on the classical criteria for apoptosis, i.e., nuclear condensation/fragmentation or membrane blebbing [30,45,56,57,58]. The characteristic morphological features of intact and apoptotic cells, as determined by inverted phase contrast microscopy, have been published [30,47,56,59,60]. At least 200 neighbouring cells from randomly selected areas were scored for the percentage of apoptotic cells at each point of measurement. Control assays ensured that the morphological features ‘nuclear condensation/fragmentation’, as determined by inverse phase contrast microscopy, were correlated to intense staining with bisbenzimide and to DNA strand breaks, detectable by the TUNEL reaction [45,47,59,60]. A recent systematic comparison of methods for the quantitation of apoptotic cells showed that there is a perfect coherence between the pattern of cells with condensed/fragmented nuclei (stained with bisbenzimide) and TUNEL-positive cells in assays with substantial apoptosis induction, whereas there was no significant nuclear condensation/fragmentation in control assays [47,56]. Further controls ensured that ROS-mediated apoptosis induction was mediated by the mitochondrial pathway of apoptosis, involving caspase-9 and caspase-3 [47,52]. The Supplementary Materials provide additional information on the characteristics of apoptosis in MKN-45 gastric carcinoma cells and on the specificity of the methods used for its determination.

### 2.4. Statistical Analysis

Statistical analysis. In all experiments, assays were performed in duplicate and standard deviations were calculated. Absence of standard deviation bars indicates that the standard deviation was too small to be reported by the graphic program. Standard deviations demonstrate reproducibility in parallel assays but do not allow statistical analysis of variance. The experiments were always performed with duplicate assays and were repeated at least twice. The Yates continuity corrected chi-square test was used for the statistical determination of significances (*p* < 0.01 = significant; *p* < 0.001 = highly significant).

## 3. Results

### 3.1. Membrane-Associated Catalase of Tumor Cells Prevents the Influx of H_2_O_2_ through Aquaporins and Subsequent H_2_O_2_-Mediated Apoptosis Induction

**Figure 1:**
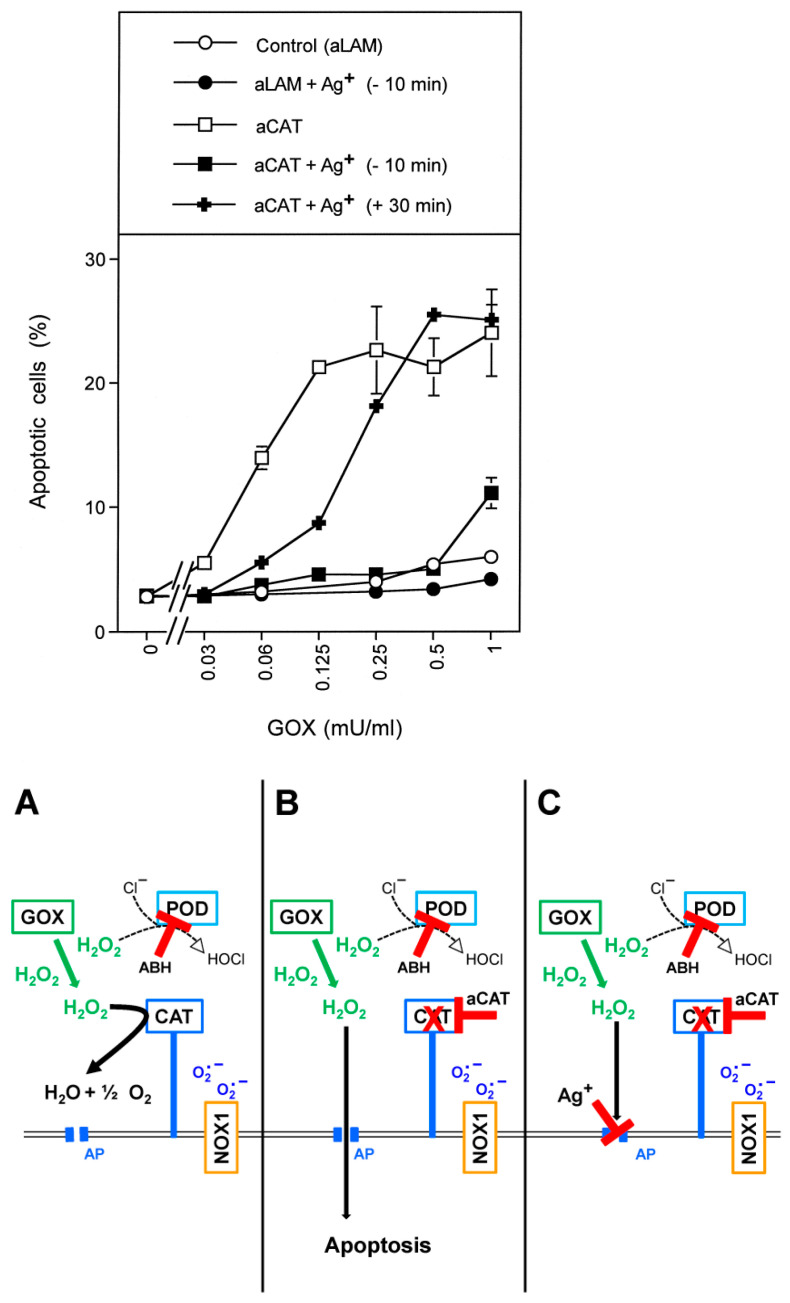
Apoptosis induction in MKN-45 gastric carcinoma cells by exogenous H_2_O_2_ is controlled by membrane-associated catalase and aquaporins. 5000 MKN-45 cells in 96 well plates with 100 µL complete medium were treated with 0.2 µg/mL control antibody (anti-laminin = aLAM) or neutralizing antibody directed towards catalase (aCAT) for 20 min at 37 °C before the indicated concentrations of H_2_O_2_-generating GOX were added. In addition, where indicated, 5 µM of aquaporin-inhibiting AgNO_3_ was added either 10 min before GOX addition (“−10 min”) or 30 min after GOX (“+30 min”). All assays were in duplicate and contained 150 µM of the peroxidase inhibitor ABH to prevent HOCl synthesis. The percentages of apoptotic cells were determined 1.5 h after addition of GOX. The results show that apoptosis induction was dependent on the concentration of GOX, required the inhibition of membrane-associated catalase (as it was effective with anticatalase but not with antilaminin) and was mediated by aquaporins (as it was inhibited by silver ions). Late addition of silver ions only prevented apoptosis induction by low concentrations of GOX. Statistical analysis: Apoptosis induction mediated by GOX concentrations of 0.19 mU/mL and higher were highly significant (*p* < 0.001). Inhibition of GOX-mediated apoptosis induction by Ag^+^ was highly significant (*p* < 0.001). The adjacent scheme to Figure 1 (below Figure 1) summarizes the principal approach and the main findings. (**A**): Membrane-associated catalase decomposes H_2_O_2_ that is generated by GOX. ABH inhibits tumor cell-derived peroxidase (POD) and thus prevents the generation of HOCl. As H_2_O_2_ is decomposed before it can enter the cells, no apoptosis is induced. (**B**): Inhibition of membrane-associated catalase by specific neutralizing antibodies allows the influx of H_2_O_2_ through aquaporins and subsequent apoptosis induction. Extracellular ROS signaling does not contribute to apoptosis induction in this experimental setting, as HOCl synthesis is prevented by ABH-mediated inhibition of peroxidase. (**C**): The role of aquaporins for influx and apoptosis induction is confirmed by inhibition of these processes by the aquaporin-inhibiting silver ions.

MKN-45 human gastric carcinoma cells at low cell density were either treated with neutralizing monoclonal antibodies directed towards catalase or irrelevant control antibodies. The aquaporin inhibitor Ag^+^ at a final concentration of 5 µM was added to one half of the assays. In order to prevent H_2_O_2_-mediated HOCl synthesis, 150 µM of the peroxidase inhibitor aminobenzoyl hydrazide (ABH) were added to all assays. This regime allowed focus explicitly on direct H_2_O_2_-mediated apoptosis induction [61]. All assays then received increasing concentrations of H_2_O_2_-generating glucose oxidase (GOX). As shown in Figure 1, tumor cells that had been treated with irrelevant control antibodies were protected towards the apoptosis-inducing effect of GOX-derived H_2_O_2_ up to a concentration of 1 mU/mL GOX. However, when their membrane-associated catalase was inhibited by neutralizing antibodies, apoptosis was induced dependent on the concentration of GOX, and finally resulting in a plateau-type of curve. GOX-mediated apoptosis induction after inhibition of catalase was prevented when aquaporins were inhibited by Ag^+^ prior to the addition of GOX. When the aquaporin inhibitor was added 30 min after, cells were no longer protected against the apoptosis-inducing effect of 0.25 mU/mL GOX and higher concentrations. However, there was still substantial protection against apoptosis induction at lower concentrations of GOX. These findings show (i) that an influx of H_2_O_2_ into the cells was required for apoptosis induction under the conditions of this experiment, (ii) that membrane-associated catalase controls the effective H_2_O_2_ level and (iii) that aquaporins control the influx of H_2_O_2_ (see the scheme to Figure 1 for summary). The data also show that the concentration of exogenous, GOX-derived H_2_O_2_ determines the time window in which aquaporins play their role in this specific experimental setting.

The previous experiment used conditions under which apoptosis was induced by H_2_O_2_ after influx into tumor cells of relatively low cell density. As recently shown [61], these conditions may lead to intracellular Fenton chemistry of H_2_O_2_. Extracellular HOCl signaling was prevented in the preceding experiment by the addition of the peroxidase inhibitor ABH and by low cell density, and therefore played no role under these conditions.

### 3.2. The Essential Cooperation between Extracellular ROS/RNS Signaling and Aquaporins

**Figure 2:**
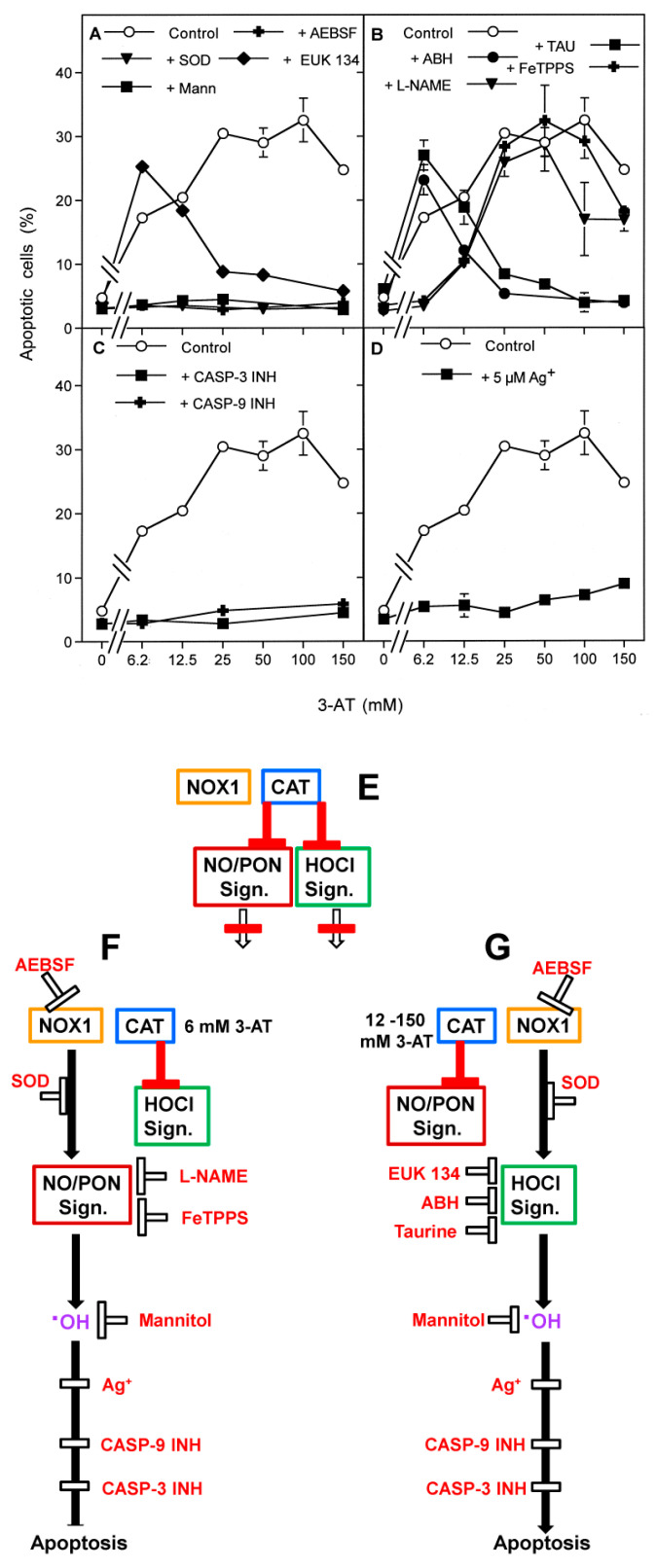
Intercellular apoptosis inducing signaling through the NO/peroxynitrite and the HOCl signaling pathways requires the cooperation with aquaporins. 12,500 MKN-45 cells in 96 well plates with 100 µL complete medium received the following inhibitors/scavengers: (**A**): 100 µM of the NOX inhibitor AEBSF, 100 U/mL MnSOD, 2 µM of the catalase mimetic EUK-8, 10 mM of the hydroxyl radical scavenger mannitol; (**B**): 50 mM of the HOCl scavenger taurine, 150 µM of the peroxidase inhibitor ABH, 20 µM of the peroxynitrite decomposition catalyst FeTPPS, 2.4 mM of the NOS inhibitor L-NAME; (**C**): 50 µM caspase-3 inhibitor, 25 µM caspase-9 inhibitor; (**D**): 5 µM AgNO_3_. Control assays remained free of inhibitors. All assays received the indicated concentrations of the catalase inhibitor 3-AT. The percentages of apoptotic cells were determined 4 h after addition of 3-AT. The result shows that inhibition of catalase was necessary for reactivation of intercellular apoptosis-inducing signaling. Apoptosis induction was dependent on superoxide anions and hydroxyl radicals at all concentrations. At low concentrations of 3-AT, apoptosis induction was mediated by the NO/peroxynitrite signaling pathway (dependency on NO synthesis, peroxynitrite, superoxide anions and hydroxyl radicals) and at higher concentrations of 3-AT the HOCl signaling pathway became responsible for apoptosis induction (dependency on H_2_O_2_, peroxidase, HOCl, superoxide anions and hydroxyl radicals). Apoptosis was dependent on caspases -9 and -3, indicating the mitochondrial pathway of apoptosis. Apoptosis induction by both signaling pathways was dependent on the action of aquaporins, as seen by the strong inhibition by Ag^+^. Statistical analysis: Apoptosis induction at all concentrations of 3-AT was highly significant (*p* < 0.001). Inhibition of 3-AT-mediated apoptosis induction by AEBSF, SOD, mannitol, caspase-3 and caspase-9 inhibitor, as well as Ag^+^, was highly significant (*p* < 0.001) at all concentrations of 3-AT. Inhibition of apoptosis by EUK-134, taurine, ABH at 3-AT concentrations of 25 mM and higher was highly significant (*p* < 0.001). Inhibition of apoptosis by L-NAME and FeTPPS at 3-AT concentrations of 6.25 and 12.5 mM was highly significant (*p* < 0.001). Scheme to Figure 2 (right side of Figure 2) summarizes the approach and the essential results described in Figure 2. (**E**): In the absence of catalase inhibitor, membrane-associated catalase efficiently prevents extracellular, NOX1-driven apoptosis-inducing signaling through the NO/peroxynitrite (NO/PON) signaling pathway and the HOCl signaling pathway. (**F**): Low concentrations of the catalase inhibitor 3-AT (6 mM) allow for selective reactivation of NO/PON signaling, which is mediated by hydroxyl radical formation and leads to apoptosis. Apoptosis induction through the NO/PON signaling pathway can be blocked by AEBSF-mediated inhibition of NOX1, scavenging of superoxide anions by SOD, inhibition of NOS by L-NAME, decomposition of peroxynitrite be FeTPPS and by scavenging hydroxyl radicals through mannitol. (**G**): At high concentrations of 3-AT (12–150 mM), HOCl signaling is reactivated on the expense of NO/PON signaling. HOCl signaling is also inhibited by AEBSF, SOD and mannitol. It is specifically inhibited by the catalase mimetic EUK-134, the peroxidase inhibitor ABH and the HOCl scavenger taurine. Both signaling pathways are strongly inhibited when the efflux of H_2_O_2_ through aquaporins is prevented by the aquaporin inhibitor Ag^+^. Both pathways are mediated by the mitochondrial pathway of apoptosis, as seen by the inhibitory effect of caspase-9 and caspase-3 inhibitors.

The next experiments focused on the role of aquaporins for apoptosis induction through extracellular apoptosis-inducing signaling, mediated by either the NO/peroxynitrite or the HOCl signaling pathway. To establish intercellular apoptosis-inducing signaling of tumor cells, membrane-associated catalase was inhibited in tumor cell populations at a cell density that was sufficiently high to allow intercellular apoptosis-inducing signaling [30]. As shown in Figure 2, increasing concentrations of the catalase inhibitor 3-aminotriazol (3-AT) caused apoptosis induction in MKN-45 cells in the mode of an optimum curve. Apoptosis induction at all concentrations of 3-AT was dependent on extracellular superoxide anions and on hydroxyl radicals, as it was inhibited by SOD, AEBSF and by mannitol. Therefore, in line with previous findings, it seems to be driven by NOX1-derived extracellular superoxide anions and finalized by hydroxyl radical-mediated lipid peroxidation [30,52]. The catalase mimetic EUK-134 inhibited apoptosis only at high concentrations of 3-AT, indicating that H_2_O_2_ was involved in apoptosis induction under the condition of efficient inhibition of membrane-associated catalase. Importantly, the effects of the other inhibitors allowed the conclusion that, under these conditions, H_2_O_2_ did not act as a direct apoptosis inducer but seemed to be the substrate for HOCl synthesis.

Apoptosis induction at low concentrations of 3-AT was inhibited by the NO synthase (NOS) inhibitor L-NAME and the peroxynitrite decomposition catalyst FeTPPS, whereas apoptosis induction at high concentrations of 3-AT was inhibited by the peroxidase inhibitor ABH and the HOCl scavenger taurine. These findings demonstrate that 3-AT induced NO/peroxynitrite signaling at low concentrations, and HOCl signaling seemed to follow and to replace NO/peroxynitrite signaling at higher concentrations of 3-AT. Both signaling pathways were finally executed by caspase-9 and caspase-3, indicating the action of the mitochondrial pathway of apoptosis. The presence of the aquaporin inhibitor Ag^+^ prevented apoptosis induction at all concentrations of 3-AT. These findings show that aquaporins have a dominant controlling function for the extracellular HOCl- and the NO/peroxynitrite-dependent apoptosis-inducing signaling pathway specifically exerted by tumor cells after inhibition of their protective membrane-associated catalase (see scheme to Figure 2 for summary). This regime, therefore, specifically addresses the biochemical interactions during novel approaches that target the distinct membrane-associated redox system of bona fide tumor cells, which are characterized by the expression of membrane-associated catalase and NOX1 [30,31]. Previous studies have established that nonmalignant cells do not respond with apoptosis induction to inhibitors or inactivators of membrane-associated catalase, as they do not express catalase on their membrane [23,30,31] and also do not express superoxide anion-generating NOX1 [11,15,16,20,21,22,23]. Please find more information on these aspects under Supplementary Materials.

### 3.3. Aquaporin Action Is Required for Apoptosis-Inducing ROS- as Well as RNS Signaling of Tumor Cells

**Figure 3:**
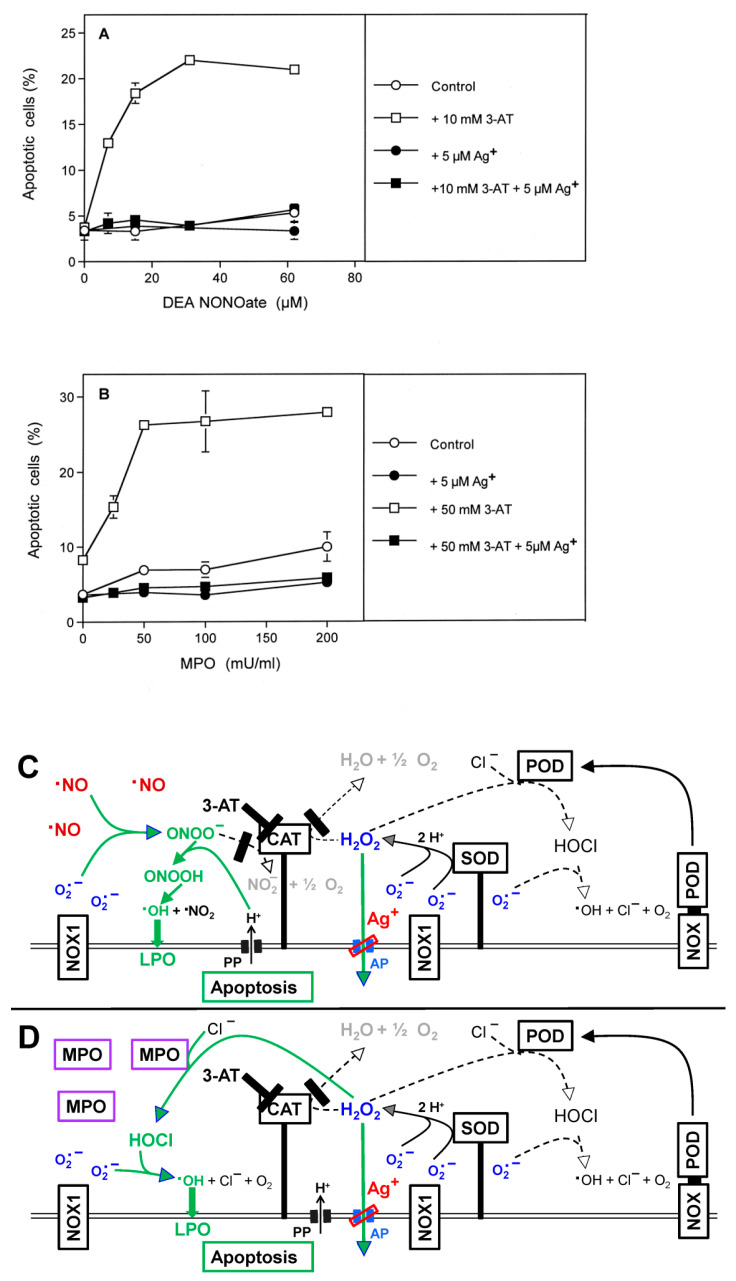
Aquaporin action is required for NO/peroxynitrite and HOCl signaling. MKN-45 gastric carcinoma cells were seeded at a density of 5000 cells (**A**) or 12 500 cells (**B**) in 96 well plates with 100 µL complete medium. Controls remained without 3-AT and Ag^+^, whereas the other assays received the indicated concentrations of 3-AT and/or Ag^+^. Assays under A received increasing concentrations of the NO donor DEA NONOate, whereas assays under B received increasing concentrations of MPO. The percentages of apoptotic cells were determined after 2.5 h (**A**) or 1.5 h (**B**). The lower cell density, 10 mM 3-AT and the NO donor DEA NONOate preferentially induced NO/peroxynitrite signaling in (**A**), whereas the higher cell density, 50 mM 3-AT and the presence of MPO fostered the HOCl pathway selectively. Apoptosis induction by both pathways was dependent on aquaporins, as it was inhibited by Ag^+^. Statistical analysis: Apoptosis induction mediated by DEAE NONOate and MPO, as well as its inhibition by Ag^+^ were highly significant (*p* < 0.001). The scheme to Figure 3 shows that selective apoptosis induction by the NO/PON signaling pathway (**C**) can be enhanced through addition of NO donors such as DEA NONOate. NO/PON signaling of tumor cells requires the presence of a catalase inhibitor (such as 3-AT) and is driven by NOX1-derived superoxide anions. Selective apoptosis induction through the HOCl signaling pathway (**D**) can be enhanced by the addition of exogenous MPO. It requires inhibition of catalase and the generation of extracellular superoxide anions by NOX1. Both pathways seem to require the parallel influx of H_2_O_2_ through aquaporins, as they are strongly inhibited by the aquaporin inhibitor Ag^+^. These data confirm the conclusions derived from the experiment described in Figure 2.

In order to focus the analysis rigidly on defined individual signaling pathways, the tumor cells were confronted with exogenously added defined signaling compounds such as the NO donor DEA NONOate or MPO at the respective optimal cell density. This approach specifically enhances NO/PON signaling or HOCl signaling (see scheme for Figure 3). For the establishment of NO/peroxynitrite signaling, the cells were seeded at a density of 50,000 cells/mL and treated with 10 mM 3-AT and increasing concentrations of the NO donor. Specific HOCl signaling was established in a cell population at a density of 125,000 cells/mL and in the presence of 50 mM 3-AT and increasing concentrations of MPO. Assays received the aquaporin inhibitor Ag^+^ or remained free of inhibitor. Control experiments confirmed that the NO donor exclusively induced NO/peroxynitrite signaling, which was inhibited by FeTPPS, but not by taurine. In contrast, MPO established HOCl signaling that was inhibited by taurine but not by FeTPPS. Figure 3A,B demonstrates that apoptosis induction through individual NO/peroxynitrite or HOCl signaling was strictly dependent on the action of aquaporins, as apoptosis induction by both individually induced pathways was completely inhibited by the aquaporin inhibitor Ag^+^.

### 3.4. Aquaporin Action Is Only Initially Required

**Figure 4:**
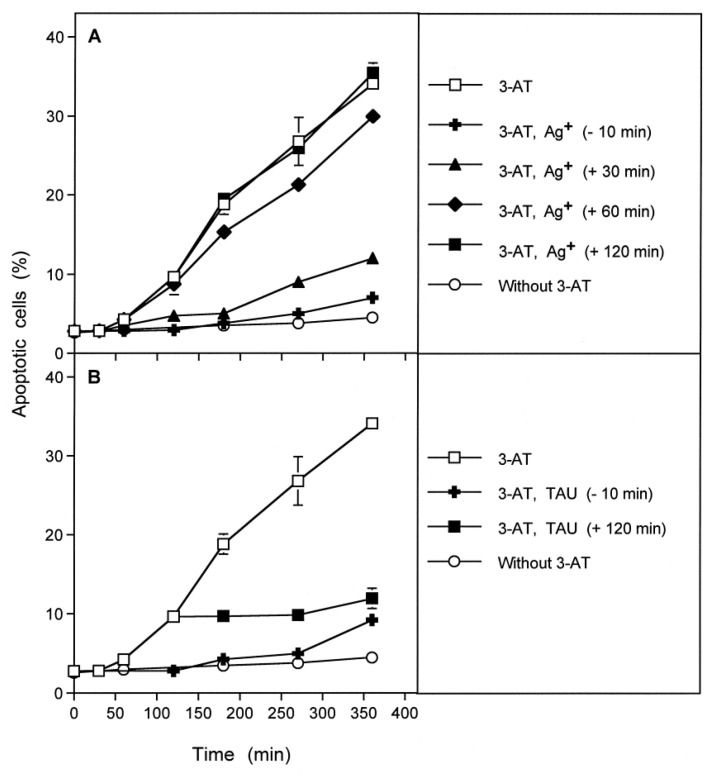

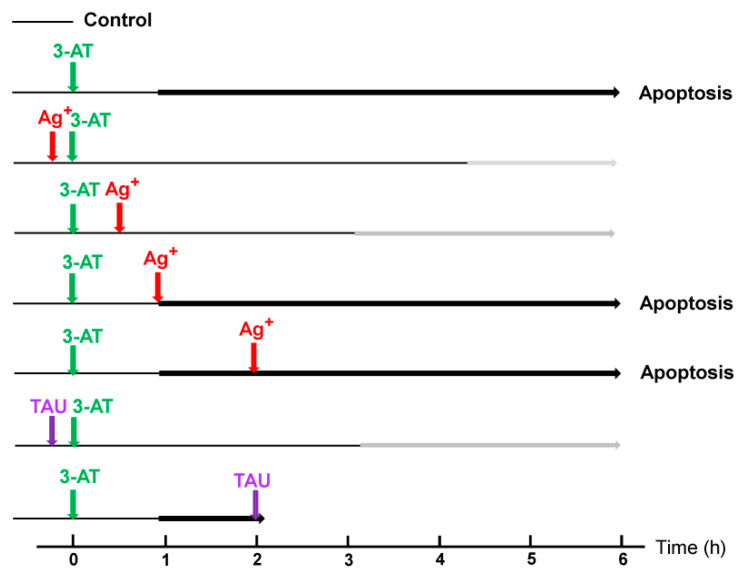
Aquaporin-mediated effects are restricted to the early phase of apoptosis induction. 12,500 MKN-45 gastric carcinoma cells were seeded in 96 well plates (100 µL medium). (**A**): Assays remained without 3-AT or received 100 mM 3-AT, where indicated. Ag^+^ was added were indicated, either 10 min before 3-AT or 30, 60 or 120 min after 3-AT. (**B**): Assays remained with 3-AT or received 100 mM 3-AT. Where indicated, the HOCl scavenger taurine was added either 10 min before or 120 min after 3-AT addition. The percentages of apoptotic cells were determined kinetically. The experiment shows that inhibition of catalase causes apoptosis induction with a lag phase of about one hour. Inhibition of aquaporins starting before the addition of 3-AT caused complete inhibition of apoptosis induction. Inhibition of aquaporins 30 min after 3-AT addition was slightly less effective and addition of the aquaporin inhibitor at 60 min caused almost no more inhibition. In contrast, addition of the HOCl scavenger taurine caused strong inhibition after addition before 3-AT or 120 min after its addition. These data show that apoptosis induction requires inhibition of catalase and the action of aquaporins during the first 60 min, i.e., during the lag phase of apoptosis induction, whereas HOCl signaling is required during the complete process of apoptosis induction. Statistical analysis. (**A**): Apoptosis induction mediated by 3-AT, as well as its inhibition by Ag^+^ added at −10 min or +30 min were highly significant (*p* < 0.001). (**B**): Inhibition of apoptosis induction by taurine was highly significant (*p* < 0.001). The scheme to Figure 4 shows that apoptosis induction in tumor cells requires inhibition of membrane-associated catalase and is characterized by a lag phase of about one hour after addition of the catalase inhibitor 3-AT. Addition of the aquaporin inhibitor Ag^+^ shortly before 3-AT or up to 30 min after its addition leads to strong inhibition of apoptosis induction, whereas the addition of Ag^+^ one or two hours after 3-AT has no effect on apoptosis induction. In contrast, addition of the HOCl scavenger taurine even two hours after 3-AT causes an immediate stop of further apoptosis induction. These data demonstrate that the aquaporin-mediated step that cooperates with HOCl signaling is confined to less than one hour after catalase inhibition, whereas HOCl signaling is contributing to signaling also at later time points.

The kinetic analysis of apoptosis induction by 100 mM 3-AT in MKN-45 cells showed that apoptosis induction started linearly after an initial lag phase of about 1 h (Figure 4A). Addition of the aquaporin inhibitor either before the catalase inhibitor or 30 min later caused nearly complete inhibition of apoptosis induction. Addition of the aquaporin inhibitor at 60 min, i.e., at the end of the lag phase, only allowed a marginal inhibitory effect. Finally, addition of the aquaporin inhibitor at 2 h after addition of 3-AT was completely ineffective. In contrast, addition of the HOCl scavenger taurine at this late time point caused an immediate stop of apoptosis induction (Figure 4B). These findings show that the aquaporin-dependent obligatory step seems to be completed during less than 60 min after addition of the catalase inhibitor, whereas the direct signaling-relevant process is continuously required thereafter, as seen by the strong inhibitory effect of the HOCl scavenger even when applied at a late time point. Please see the scheme to Figure 4 for summary.

### 3.5. H_2_O_2_ Intruding through Aquaporins Is the Dismutation Product of NOX1-Derived Extracellular Superoxide Anions

The data obtained so far indicate that intercellular apoptosis-inducing signaling of tumor cells after inhibition of their protective catalase seemed to require an influx of H_2_O_2_ through aquaporins to sensitize the cells to the apoptosis-inducing effect of signaling-derived hydroxyl radicals. This sensitizing step seems to require approximately one hour. It therefore well explains the lag phase of apoptosis induction after addition of the catalase inhibitor.

**Figure 5:**
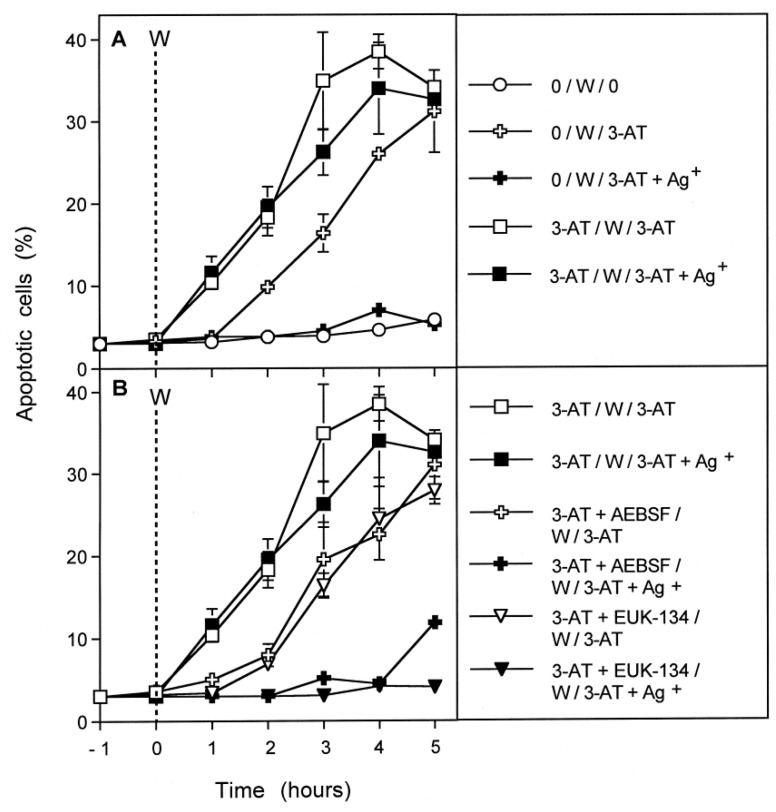

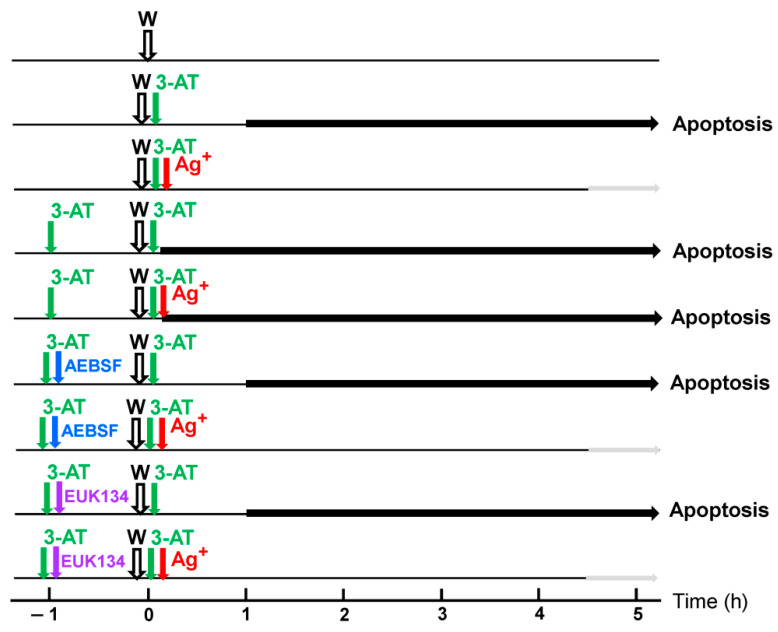
The sensitizing effect mediated by aquaporins requires extracellular H_2_O_2_ generated through dismutation of NOX1-derived superoxide anions. The principle of this experiment was the division of treatment of the cells in a pretreatment time of 1 h (−1 until 0 h in the graph) followed by three washing steps (“W”) and subsequent continuation of treatment in 96 well plates (12,500 cells/assay). Compounds present during pretreatment or after the washing step are indicated in the legend incorporated into the graph. The following concentrations were used: 3-AT: 100 mM; Ag^+^: 5 µM; AEBSF: 100 µM; EUK-134: 20 µM. The percentages of apoptotic cells were determined kinetically. (**B**): The data confirm that catalase inhibition is necessary for apoptosis induction. Pretreatment without 3-AT, followed by 3-AT treatment after the washing step (0/W/3-AT) resulted in an apoptosis kinetics that required one hour lag phase after the washing step and that was completely blocked when Ag^+^ had been added together with 3-AT (0/W/3-AT + Ag^+^). When 3-AT was present during preincubation as well as after the washing step (3-AT/W/3-AT), apoptosis induction started immediately after the washing step, requiring no further lag phase in addition to pretreatment. Addition of Ag^+^ after one hour of pretreatment (3-AT/W/3-AT + Ag^+^) did not block apoptosis induction, indicating that the aquaporin-mediated step had been active and sufficient during the 1 h pretreatment with 3-AT. (**B**): The data show that the presence of the NOX1 inhibitor AEBSF or the catalase mimetic EUK-134 during the 1 h pretreatment with 3-AT prevented the aquaporin-mediated effect. Taken together, the data from (**A**,**B**) show that NOX- derived superoxide anions and their dismutation product H_2_O_2_ are necessary for the aquaporin-mediated effect that took approximately one hour and that preceded the onset of apoptosis induction through catalase inhibition. Statistical analysis: The effects described under (**A**,**B**) were highly significant (*p* < 0.001). The scheme to Figure 5 illustrates that catalase inhibition by 3-AT led to apoptosis induction after a lag phase of about one hour. Inhibition of aquaporin function by Ag^+^ at the same time as catalase inhibition completely prevented apoptosis induction. Addition of 3-AT for one hour, followed by a washing step (“W”) and further addition of 3-AT led to apoptosis induction without lag phase after second addition of 3-AT. Addition of Ag^+^ at the second time point of addition of 3-AT no longer blocked apoptosis induction, confirming that the aquaporin-dependent step was restricted to the time between first and second addition of 3-AT, i.e., less than one hour. When the NOX1 inhibitor AEBSF or the catalase mimetic EUK-134 were added during the first hour of presence of 3-AT and then these inhibitors were washed away, a lag phase of one hour was seen after second addition of 3-AT and sensitivity to the inhibitory function of Ag^+^ was resumed. These data show that the aquaporin-mediated step requires extracellular cellular superoxide anion generation and the presence of the dismutation product H_2_O_2_.

In the preceding experiments, the most likely source for H_2_O_2_ that had to pass aquaporins in order to achieve the sensitizing effect, was the dismutation of NOX1-derived superoxide anions. To elucidate this biochemical scenario, MKN-45 tumor cells were pretreated with the catalase inhibitor 3-AT for one hour in the absence or presence of either the NOX1 inhibitor AEBSF or the catalase mimetic EUK-134. In parallel, cells were pretreated for the same time in the absence of 3-AT, for control. After pretreatment, cells were washed to remove all inhibitors, and assays were further cultivated in the presence or absence of 3-AT. As shown in Figure 5A, pretreatment with 3-AT for one hour allowed immediate onset of apoptosis induction after renewed addition of the catalase inhibitor after the washing step. This response was independent of aquaporins, as seen from the lack of effect of the aquaporin inhibitor Ag^+^. In contrast, cells that had been incubated in the absence of 3-AT for one hour required one hour lag phase before they responded to the catalase inhibitor that had been added after the washing step. Importantly, the cells in these assays did not show apoptosis induction in the presence of Ag^+^, indicating the role of aquaporins in this experimental setting. When, however, pretreatment with 3-AT had been performed either in the presence of the NOX1 inhibitor AEBSF or the catalase mimetic EUK-134, the cells responded to renewed addition of 3-AT analogously to those that had not been pretreated with 3-AT (Figure 5B). They showed a lag phase of one hour and their response was completely inhibited by Ag^+^. These data show that after inhibition of tumor cell protective catalase, the dismutation product of NOX1-derived superoxide anions, i.e., H_2_O_2_, is required to enter the tumor cells through aquaporins and to cause the sensitization for apoptosis-inducing signaling. This sensitizing step seemed to be completed within one hour, whereas subsequent apoptosis-inducing signaling itself was ongoing for several hours. Please find the principle of this experiment and its major conclusions in the scheme to Figure 5.

### 3.6. Targeting of Glutathione/Glutathione Peroxidase by Intruding H_2_O_2_

Based on these initial findings and on the established literature on the role of glutathione peroxidase 4/glutathione as antagonists of lipid peroxidation-mediated apoptosis induction [62], the following working hypothesis was established. It was assumed that the aquaporin-mediated influx of H_2_O_2_ after catalase inhibition might cause a depletion of intracellular glutathione. This would then abrogate the interference of GPX4/GSH with apoptosis induction by lipid peroxidation, through hydroxyl radicals derived from the NO/peroxynitrite and the HOCl signaling pathway.

**Figure 6:**
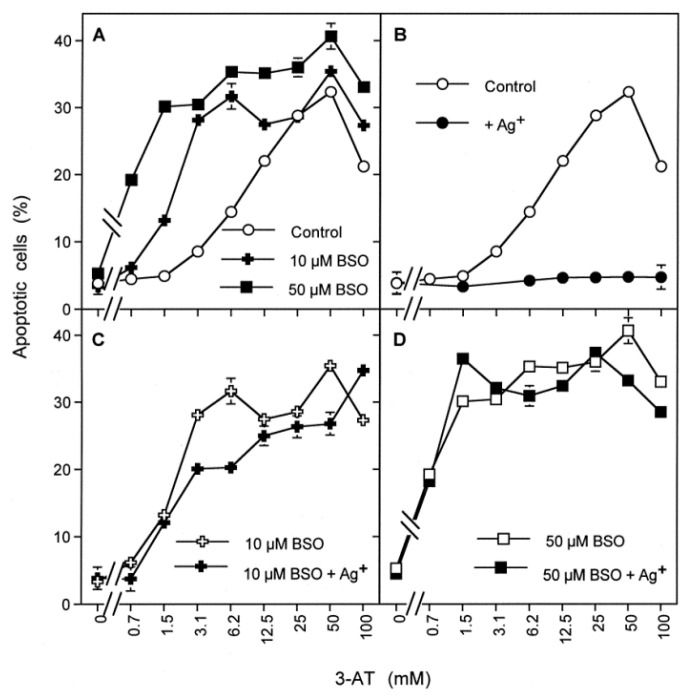

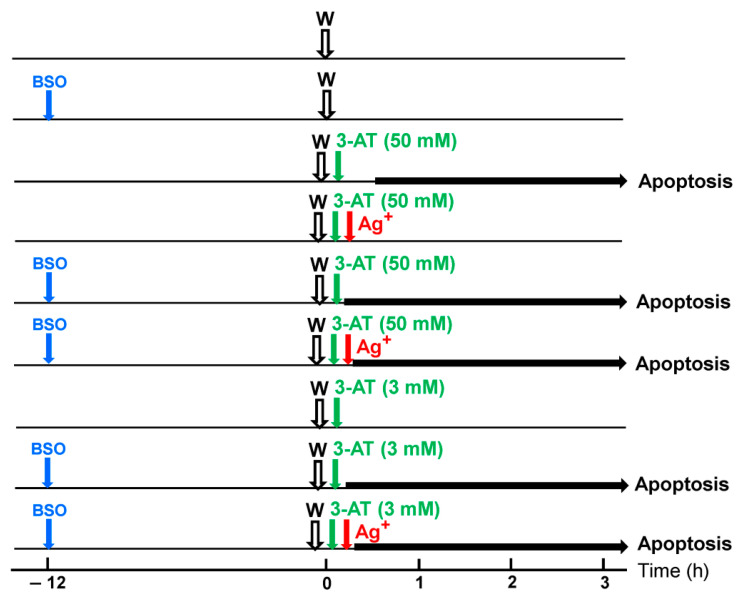
Pretreatment with the glutathione synthase inhibitor BSO substitutes for the effect of aquaporins. MKN-45 cells (125,000 cells/mL) were pretreated without BSO (control) or in the presence of 10 µM or 50 µM BSO for 12 h at 37 °C. The cells were washed and seeded in 96 well plates (12,500 cells per assay). Where indicated, 5 µM Ag^+^ was added after seeding in the 96 well plates. The percentages of apoptotic cells were determined after 3 h in BSO pretreated cultures and 4.5 h in controls. (**A**): The data show that BSO-pretreatment is not sufficient for apoptosis induction alone but increases the efficiency of low concentrations of 3-AT, but not of high concentrations. (**B**): Apoptosis induction in control cells is dependent on inhibition of catalase and on the action of aquaporins, as it requires inhibition of catalase and is completely blocked by Ag^+^. (**C**,**D**): BSO-pretreated cells require catalase inhibition for apoptosis induction in a process that is independent of the action of aquaporins, as seen by the lack of inhibition by Ag^+^. These data show that the effects of aquaporin action can be explained by glutathione depletion. Statistical analysis. (**A**): The enhancing effect of BSO pretreatment at low concentrations of 3-AT were highly significant (*p* < 0.001). (**B**): Inhibition by Ag^+^ was highly significant (*p* < 0.001). (**C**,**D**): The lack of inhibition by Ag^+^ after BSO pretreatment was highly significant (*p* < 0.001). The scheme to Figure 6 illustrates that the tumor cells do not show apoptosis in the absence of catalase inhibitor, even if they have their intracellular glutathione level has been lowered by BSO treatment. Addition of 50 mM 3-AT leads to apoptosis induction characterized by a lag phase and dependence on aquaporin action, as it is inhibited by Ag^+^. Pretreatment with BSO, followed by the addition of 50 mM 3-AT leads to apoptosis induction without a lag phase and independent of aquaporin function. This finding shows that glutathione depletion substitutes for the aquaporin effect. 3 mM 3-AT was not sufficient for apoptosis induction in control cells but allowed apoptosis induction in BSO-pretreated cells without a lag phase and independent of aquaporin function, indicating that BSO treatment sensitized the cells for apoptosis induction and substituted for the aquaporin effect.

If this working hypothesis were correct, pretreatment of MKN-45 tumor cells with BSO, an inhibitor of glutathione synthase should allow subsequent ROS/RNS-dependent apoptosis induction after catalase inhibition, even in the presence of aquaporin inhibitors that prevented the influx of extracellular H_2_O_2_. To address this question, MKN-45 cells were pretreated with BSO, and their response to inhibition of catalase and aquaporins was compared to control cells. As shown in Figure 6A, BSO-pretreatment under the conditions chosen was not sufficient to cause apoptosis induction by itself, but it enhanced the apoptosis-inducing potential of low concentrations of the catalase inhibitor 3-AT, establishing a synergistic effect.

**Figure 7:**
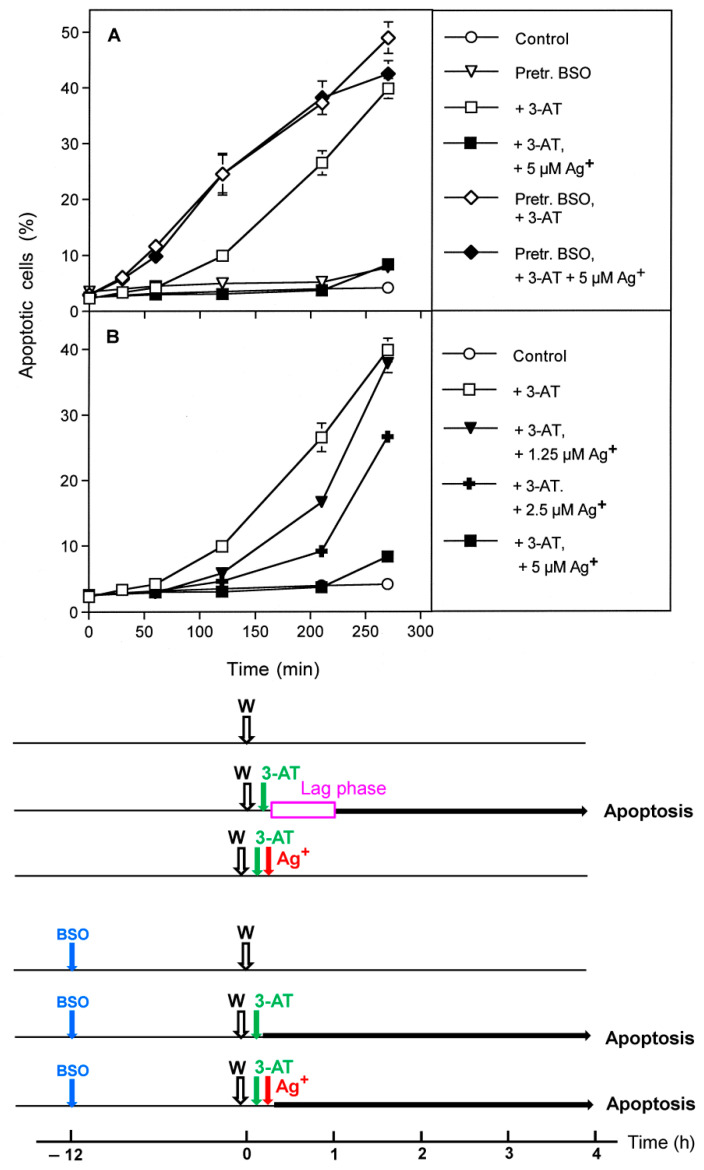
Kinetic analysis of the effects of glutathione depletion and aquaporin action. MKN-45 cells were pretreated for 14 h at 37 °C either in the absence of BSO (control) or in the presence of 10 µM BSO. Preincubation was followed by three washing steps and seeding of the cells into 96 well plates (12,500 cells/assay). (**A**): Control cells and BSO-pretreated cells received either no further addition, or 100 mM 3-AT (3-AT) or 100 mM 3-AT plus 5 µM Ag^+^. The kinetics of apoptosis induction were determined. The results confirmed that BSO-pretreatment by itself was not sufficient for apoptosis induction. Apoptosis induction in control cells required a lag phase of about one hour and was completely dependent on aquaporins, as seen by the strong inhibition by Ag^+^. In contrast, BSO-pretreated cells did not require a lag phase for apoptosis induction and were independent of the action of aquaporins. These data confirm that the lag phase is due to the action of aquaporins and that glutathione depletion by BSO pretreatment substitutes for the aquaporin effect. (**B**): Control cells remained, without further addition, or received 100 mM 3-AT in the absence of presence of increasing concentrations of Ag^+^. Apoptosis induction was determined kinetically and illustrates the dependency of inhibition of the aquaporin-mediated effect on the concentration of Ag^+^. Statistical analysis. (**A**): The effects of BSO pretreatment on the kinetics of apoptosis induction and dependence on aquaporins were highly significant (*p* < 0.001). (**B**): The dependency of the inhibitory potential of Ag^+^ on its concentration was highly significant (*p* < 0.001). The Scheme to Figure 7 illustrates that 3-AT causes apoptosis induction in tumor cells, characterized by a lag phase and dependence on the action of aquaporins. BSO pretreatment for 12 h is not sufficient for apoptosis induction but allows for apoptosis induction by 3-AT without a lag phase and independent of aquaporins. These findings are in line with the concept that glutathione depletion sensitizes the tumor cells for the effect of ROS signaling, without the need for additional function of aquaporins.

Whereas apoptosis induction in control cells was completely blocked by the aquaporin inhibitor Ag^+^ (Figure 6B), apoptosis induction in BSO-pretreated cells was only marginally or not all affected by Ag^+^ (Figure 6C,D). These findings show that apoptosis induction in BSO-pretreated tumor cells still required inhibition of catalase but was independent of the influx of H_2_O_2_ through aquaporins. A kinetic analysis confirmed that apoptosis induction by 3-AT in control cells started after a lag phase of one hour, whereas apoptosis induction in BSO-pretreated cells started immediately after addition of the catalase inhibitor (Figure 7A). Whereas apoptosis induction in control cells was completely inhibited by the aquaporin inhibitor Ag^+^, apoptosis induction in BSO-pretreated cells was not (Figure 7A), indicating that BSO-pretreatment had substituted for the controlling effect of aquaporins.

Complete inhibition of apoptosis induction in control cells required a concentration of 5 µM Ag^+^ (Figure 7B). A gradual decrease in the concentration of Ag^+^ resulted in a decrease of inhibition, and an extension of the lag phase resulting in a delayed expression of apoptosis induction (Figure 7B).

**Figure 8:**
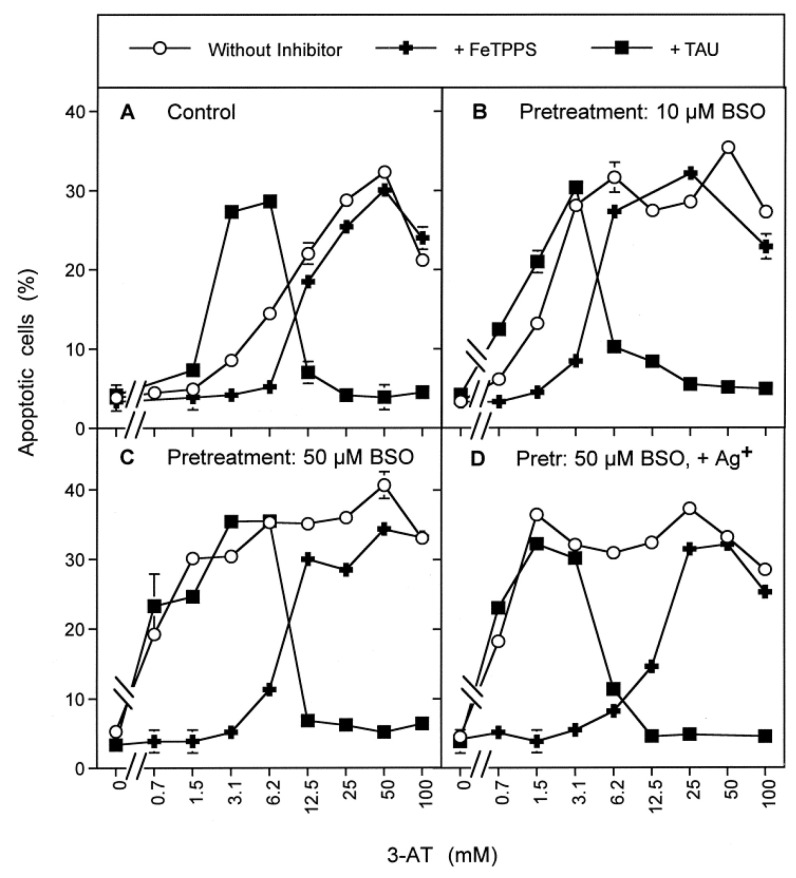

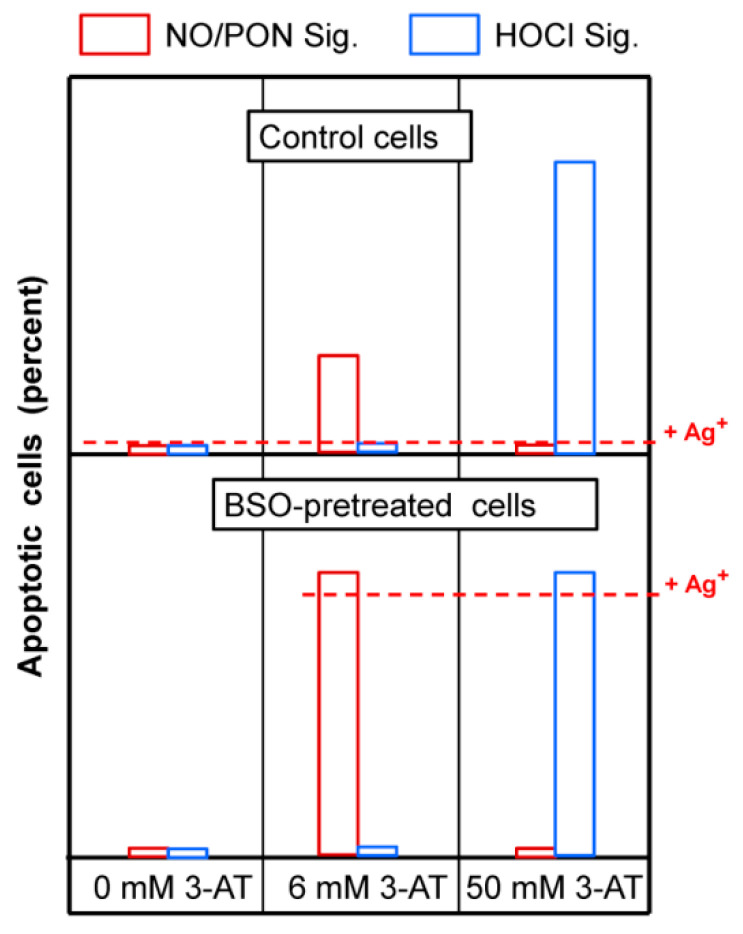
Apoptosis induction after glutathione depletion is dependent of catalase inhibition and intercellular ROS/RNS signaling, but independent on aquaporin action. MKN-45 cells were pretreated for 12 h at 37 °C in the absence of BSO (“control”) or in the presence of 10 µM or 50 µM BSO. After preincubation, cells were washed and seeded in 96 well plates (12,500 cells/assay). Control cells (**A**), cells pretreated mit 10 µM BSO (**B**), cells pretreated with 50 µM BSO (**C**) and cells pretreated with 50 µM BSO, plus addition of 5 µM Ag^+^ after pretreatment (**D**) remained without inhibitors or received either 20 µM of the peroxynitrite decomposition catalyst FeTPPS or 50 mM of the HOCl scavenger taurine. 3-AT at the indicated concentrations was added to all assays and the percentages of apoptotic cells were determined after 4.5 h (**A**) or 3 h (**B**–**D**). The data show that 3-AT-dependent apoptosis induction in control cells and BSO-pretreated cells was due to initial NO/peroxynitrite signaling at lower concentrations of 3-AT, followed by HOCl signaling at higher concentrations. It is also seen that the enhanced apoptosis induction in BSO-pretreated cells at the left shoulder of the curve was essentially due to enhancement of NO/peroxynitrite signaling. Statistical analysis. Differential inhibition of NO/peroxynitrite and HOCl signaling by FeTPPS and taurine was highly significant (*p* < 0.001). The scheme to Figure 8 illustrates that 6 mM 3-AT preferentially reactivates NO/PON signaling, whereas 50 mM 3-AT cause apoptosis induction through the HOCl signaling pathway. BSO pretreatment of the tumor cells does not allow for apoptosis induction in the absence of the catalase inhibitor but enhances NO/PON-mediated apoptosis induction and renders 3-AT-mediated apoptosis induction independent of aquaporin action.

The central question to be clarified was whether apoptosis induction through inhibition of catalase in BSO-pretreated cells still required complex intercellular ROS/RNS signaling, or whether an influx of H_2_O_2_ was sufficient for apoptosis induction in glutathione-depleted cells. The results presented in Figure 8 show that apoptosis induction in BSO-pretreated cells, as well as in BSO-untreated cells, was always mediated by extracellular ROS/RNS signaling. Thereby, NO/peroxynitrite signaling was followed by HOCl signaling (Figure 8). This finding demonstrates that though glutathione depletion efficiently sensitizes the tumor cells for apoptosis induction, the central extracellular apoptosis-inducing principles are the same for glutathione-depleted and control cells, and the role of intruding H_2_O_2_ seems to be restricted to the sensitizing step.

### 3.7. Dominant Role of Aquaporins after Singlet Oxygen-Mediated Inactivation of Membrane-Associated Catalase

**Figure 9:**
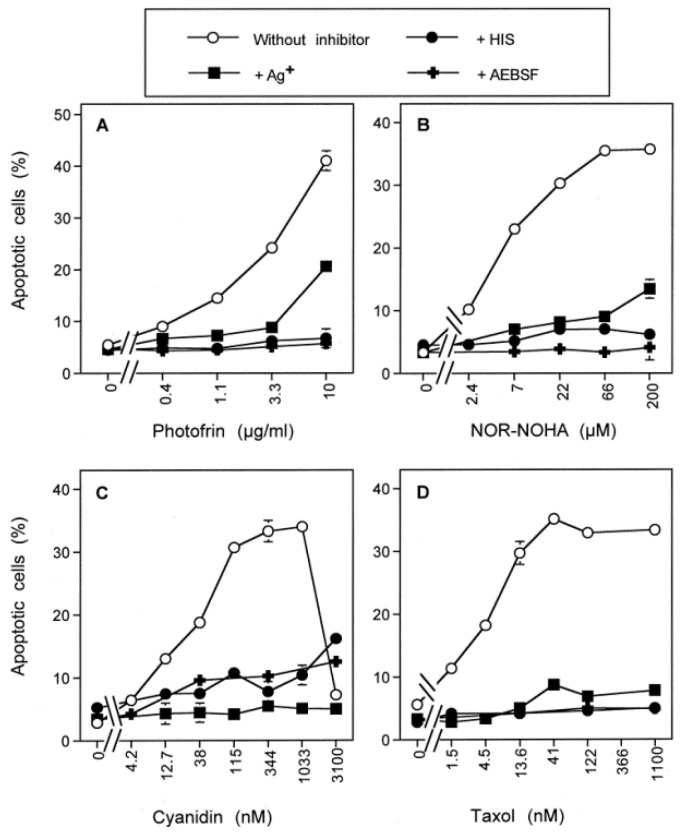
Central role of aquaporin-mediated cooperation for intercellular ROS/RNS signaling after catalase inactivation. 12,500 MKN-45 cells per assay (96 well tissue culture cluster) were treated with either the indicated increasing concentrations of the photosensitizer photofrin (**A**), the arginase inhibitor NOR-NOHA (**B**), cyanidin (**C**) or taxol (**D**). The assays remained either without inhibitors or received 2 mM of the singlet oxygen scavenger histidine (“+HIS”), the aquaporin inhibitor Ag^+^, or the NOX1 inhibitor AEBSF. Assays under (**A**) were first illuminated at room temperature with visible light for 20 min and then further incubated for 3.5 h before the percentages of apoptotic cells were determined. Assays under (**B**–**D**) were incubated for 3.5 h and then the percentages of apoptotic cells were determined. The results confirm that direct treatment with singlet oxygen (**A**) or generation of tumor cell-derived singlet oxygen after modulation the endogenous NO level (Bauer, 2015) caused apoptosis induction. In all four systems, apoptosis induction was dependent on the action of singlet oxygen, the activity of NOX1 and the action of aquaporins. These findings demonstrate that the concept of aquaporin-mediated cooperation with ROS-mediated apoptosis-inducing signaling is relevant for a broad variety of treatments that are based on inactivation of tumor cell protective catalase. Statistical analysis. Apoptosis induction by the indicated inducers, as well as inhibition of apoptosis induction by AEBSF, histidine and Ag^+^ were highly significant (*p* < 0.001).

The membrane-associated catalase of tumor cells can be inhibited by compounds such as 3-AT [30] or can be inactivated by direct application of singlet oxygen [51], singlet oxygen generation by physical plasma or plasma-activated medium [38,39,40,41] or by singlet oxygen generation after modulation of the endogenous NO level [47,50]. These modes of treatment have in common that they are related to singlet oxygen-dependent catalase inactivation, followed by autoamplificatory generation of secondary singlet oxygen. This causes an optimal degree of catalase inactivation and allows for subsequent establishment of intercellular apoptosis-inducing signaling [30,38,39,40,41,47,50,51,63]. Figure 9 shows that apoptosis induction in MKN-45 cells by direct application of the classical photosensitizer photofrin, by the arginase inhibitor NOR-NOHA or the NOD inhibitors cyanidin and taxol was uniformly (i) characterized by dependence of apoptosis induction on the concentration of the respective agent, (ii) dependent on NOX1 activity, and (iii) mediated by singlet oxygen. The action of each of these antitumor agents in addition required the activity of aquaporins, indicating that this control level is in general inherent to apoptosis induction that utilizes the reactivation of intercellular ROS/RNS-driven apoptosis induction selectively in tumor cells.

### 3.8. The Role of the xC Transporter for the Control of Apoptosis Induction

Preceding experiments showed that glutathione depletion through inhibition of glutathione synthesis [64] abrogated the need for aquaporin-mediated influx of H_2_O_2_ into cells for optimal apoptosis induction. Inhibition of the xC transporter by sulfasalazine is an alternative mode to lower the intracellular level of glutathione [42]. If our conclusions based on the results obtained with the glutathione synthase inhibitor BSO were correct, sulfasalazine treatment of tumor cells should cause of the same effect as BSO.

**Figure 10:**
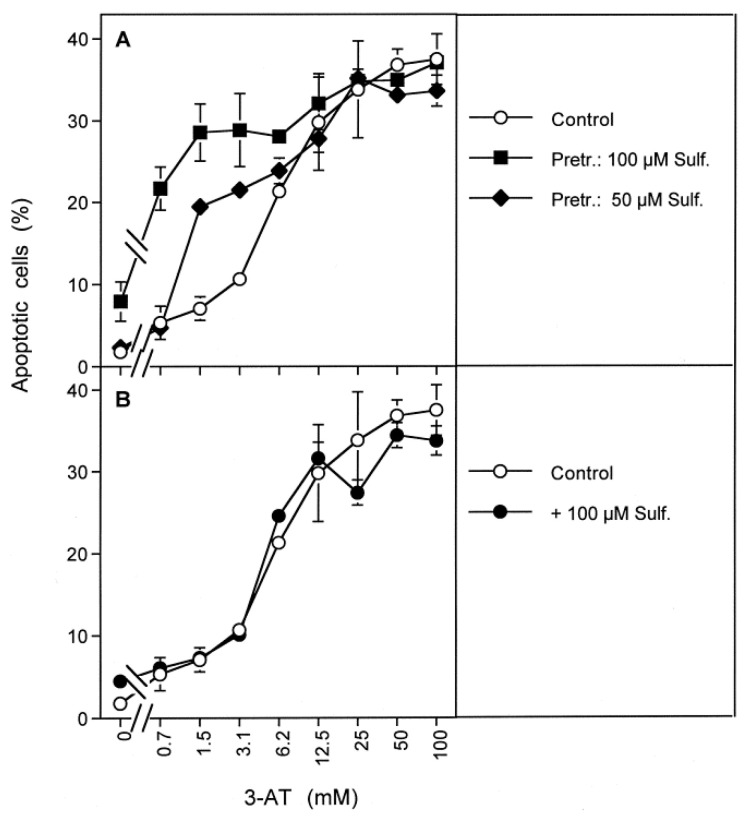
Pretreatment of tumor cells with the xCT inhibitor sulfasalazine enhances apoptosis induction after application of low concentrations of 3-AT. MKN-45 cells (200,000 cells/mL) were pretreated for 14 h in the absence of sulfasalazin (control) or presence of 50 µM or 100 µM sulfasalazine. After pretreatment, cells were washed and reseeded at a density of 12,500 cells/well in 96 well tissue culture clusters. (**A**): Control cells and cells pretreated with 50 µM or 100 µM sulfasalazine received the indicated concentrations of 3-AT. (**B**): Control cells remained without further addition or received 100 µM sulfasalazine immediately before the application of 3-AT. Assays as described under (**A**,**B**) were incubated for 5 h and the percentages of apoptotic cells were determined. The results show that pretreatment with sulfasalazine caused an enhancement of apoptosis induction in the low concentration range of 3-AT, but not at higher concentrations of 3-AT. Addition of sulfasalazine together with 3-AT had no enhancing effect. Statistical analysis. The enhancement of 3-AT-mediated apoptosis induction through pretreatment with sulfasalazine was highly significant (*p* < 0.001), whereas there was not significant effect of sulfasalazine added together with 3-AT.

Pretreatment of MKN-45 cells with increasing concentrations of sulfasalazine for 14 h caused a concentration-dependent sensitization for apoptosis induction by low concentrations of 3-AT (Figure 10A). The presence of sulfasalazine during treatment with 3-AT without previous pretreatment was not sufficient to induce this enhancing effect (Figure 10B).

**Figure 11:**
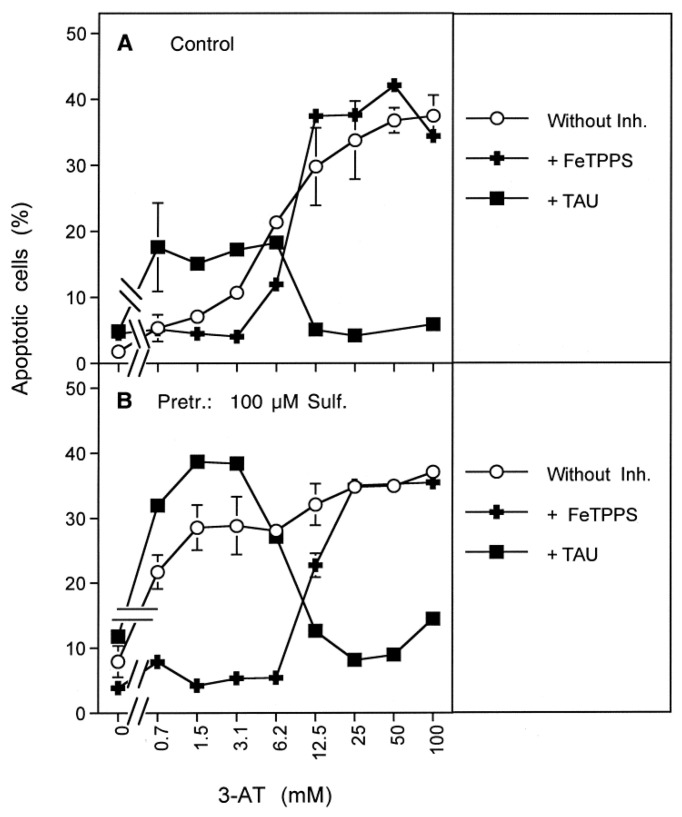
Quality of ROS/RNS signaling in controls and after sulfasalazine pretreatment. MKN-45 cells (200,000 cells/mL) were pretreated for 14 h in the absence of sulfasalazin (control) (**A**) or presence of 100 µM sulfasalazine (**B**). After pretreatment, cells were washed and reseeded at a density of 12,500 cells/well in 96 well tissue culture clusters. Assays were kept without additional inhibitors or in the presence of 20 µM FeTPPS or 50 mM taurine. 3-AT was added at the indicated concentrations and the percentages of apoptotic cells were determined after 5 h incubation. At concentrations of 3-AT of 12.5 mM and higher, HOCl signaling prevailed in the assays described in (**A**,**B**). At lower concentrations of 3-AT, NO/peroxynitrite signaling was dominant. Pretreatment with sulfasalazine caused an enhancement of NO/peroxynitrite signaling. Statistical analysis. The effect of sulfasalazine pretreatment on apoptosis induction by low concentrations of 3-AT was highly significant (*p* < 0.001). The differential inhibition by FeTPPS and taurine was highly significant (*p* < 0.001).

Increasing concentrations of 3-AT caused apoptosis induction by NO/peroxynitrite signaling at lower concentrations of 3-AT, both in control tumor cells and in tumor cells that had been pretreated with sulfasalazine, as seen by the strong inhibitory effect of FeTPPS and the lack of inhibition of taurine. At higher concentrations of 3-AT, apoptosis induction in pretreated as well as control cells was dependent on HOCl signaling, as seen by the reverse inhibitor profile (Figure 11). The observed synergistic effect between sulfasalazine pretreatment and catalase inhibition seemed to be mainly determined by NO/peroxynitrite signaling (Figure 11B).

**Figure 12:**
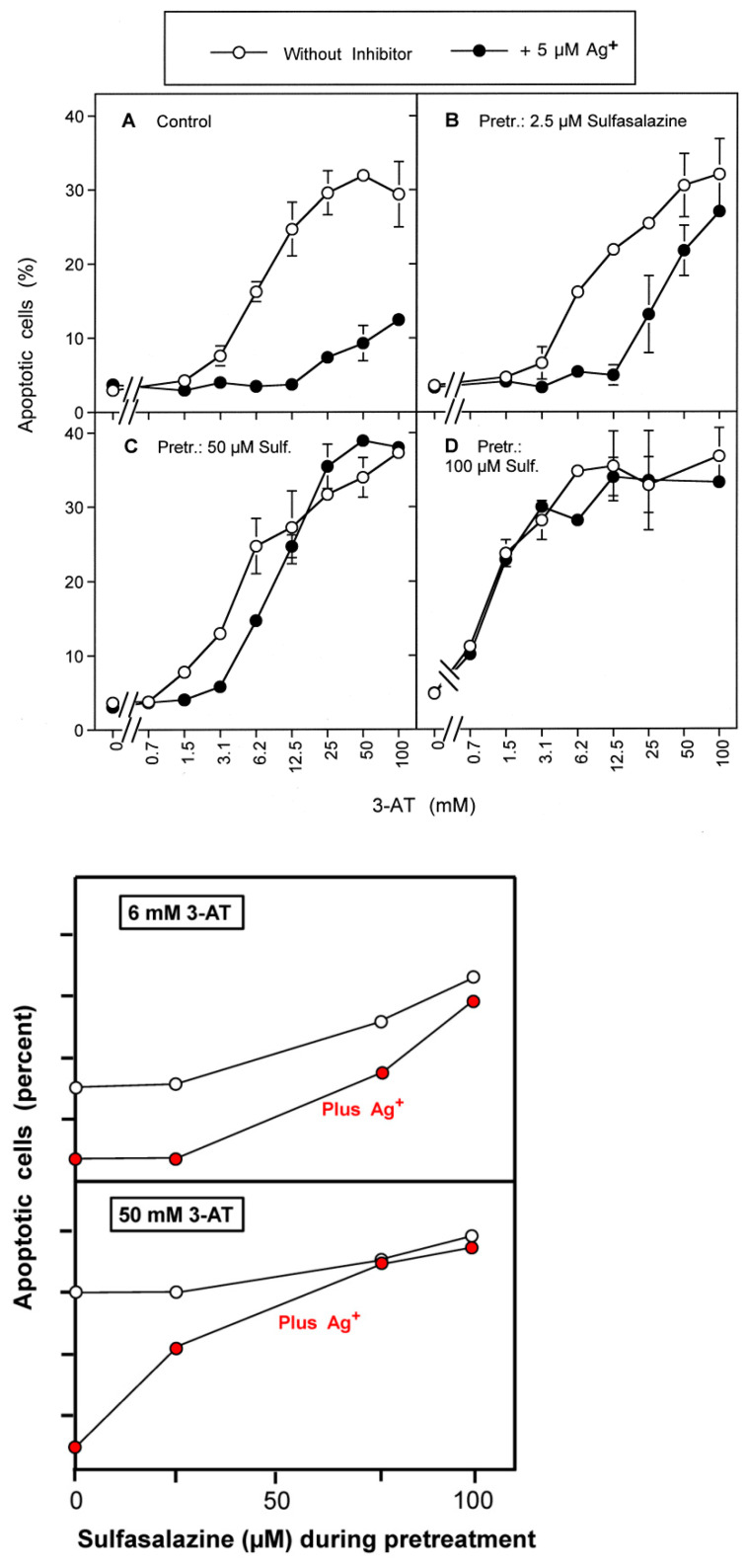
Sulfasalazine pretreatment renders ROS/RNS-dependent apoptosis-inducing signaling independent of the action of aquaporins. MKN-45 cells (200,000 cells/mL) were pretreated for 14 h in the absence of sulfasalazin (control) (**A**) or presence of increasing concentrations of sulfasalazine (**B**: 2.5 µM; **C**: 50 µM; **D**: 100 µM). After pretreatment, cells were washed and reseeded at a density of 12,500 cells/well in 96 well tissue culture clusters. Assays were kept either without additional inhibitors or in the presence of 5 µM Ag^+^. The percentages of apoptotic cells were determined after 5 h. The data show that apoptosis induction in control cells was strongly dependent on the action of aquaporins, whereas the preincubation with increasing concentrations of sulfasalazine caused a decreased dependence on aquaporins. Cells that had been pretreated with 100 µM sulfasalazine were completely independent of the action of aquaporins. Statistical analysis. The gradual differences in inhibition by Ag^+^, depending on the concentration of sulfasalazine during pretreatment were highly significant (*p* < 0.001). The scheme to Figure 12 summarizes that sulfasalazine pretreatment renders 3-AT-mediated apoptosis induction independent of the action of aquaporins. This effect is dependent on the concentration of sulfasalazine during pretreatment. The scheme also indicates that apoptosis induction mediated by 6 mM 3-AT is significantly enhanced by sulfasalazine pretreatment.

3-AT-mediated apoptosis induction in control cells was strongly inhibited by the aquaporin inhibitor Ag^+^ (Figure 12A), whereas apoptosis induction in cells pretreated with 100 µM sulfasalazine was not affected at all by Ag^+^ (Figure 12D). In tumor cells that had been pretreated with intermediary concentrations of sulfasalazine (25 µM, 50 µM), the inhibitory effect of Ag^+^ was also intermediary (Figure 12B,C). Thereby, inhibition by Ag^+^ was more prominent in the lower concentration range of 3-AT and the range of 3-AT that allowed inhibition by sulfasalazine became lower with an increasement of the sulfasalazine concentration.

These results show that depletion of the intracellular glutathione level through sulfasalazine-mediated inhibition of the xC transporter abrogates the need for aquaporin-mediated sensitization of the tumor cells for apoptosis induction through hydroxyl radical-dependent lipid peroxidation. These data confirm the data obtained in experiments that utilized the effect of the glutathione synthase inhibitor BSO and therefore confirm the central function of intracellular glutathione for the counteraction towards the apoptosis-inducing effects of extracellular RNS/ROS signaling.

## 4. Discussion

Two substantially different approaches were used in this study to elucidate the interplay between aquaporins and apoptosis induction by extracellular ROS/RNS. Approach A focused on apoptosis induction in tumor cells after inhibition of membrane-associated catalase and addition of excess exogenous H_2_O_2_. Approach B utilized the specific chemical biology of tumor cells that reactivate intercellular NO/peroxynitrite or HOCl signaling after inhibition of membrane-associated catalase [30,31].

Approach A was performed by abrogating the resistance of tumor cells towards exogenous H_2_O_2_ through application of neutralizing antibodies directed towards membrane-associated catalase, followed by addition of H_2_O_2_-generating glucose oxidase (GOX). The relatively low cell density and the presence of the peroxidase inhibitor ABH thereby prevented the establishment of HOCl signaling, whereas the applied relatively high concentrations of GOX ensured direct apoptosis induction by H_2_O_2_. Intracellular catalase, which was not reached by the cell-impermeable antibodies obviously played no protective role towards exogenous H_2_O_2_, in line with a previous report [31]. As, in this setting, apoptosis induction by GOX-derived H_2_O_2_ was strictly dependent on the parallel inhibition of membrane-associated catalase, the dominant control of H_2_O_2_ by membrane-associated catalase was confirmed [30,31]. As pretreatment of the cells with aquaporin-inhibiting silver ions prevented apoptosis induction, the role of the aquaporins for the entry process of H_2_O_2_ into the cells was also ensured. These data show the tight connection between the control function of membrane-associated catalase and aquaporins, resulting in striking cooperativity: membrane-associated catalase controlled the concentration of available H_2_O_2,_ whereas aquaporins ensured the entry of H_2_O_2_ into the cells. The differential effect of silver ions added 10 min before or 30 min past GOX, sheds light on the dynamics and concentration dependencies of the underlying processes. The results obtained through approach A also confirmed the analytical strength of catalase neutralizing antibodies and of the aquaporin inhibitor Ag^+^. This was the basis for the inhibitor-based analysis of the more complex intercellular NO/peroxynitrite and HOCl signaling and its control by membrane-associated catalase and intracellular glutathione/glutathione peroxidase.

Approach B utilized the reactivation of intercellular NO/peroxynitrite or HOCl signaling after inhibition of tumor cell membrane-associated catalase by 3-AT [30,31]. Both pathways are driven by extracellular superoxide anions generated by membrane-associated NOX1, a hallmark of malignant cells [19,20,21,22,23,24]. The specificity of the pathways is either determined by NOS-derived NO, leading to NO/peroxynitrite signaling, or by the extracellular peroxidase domain shed from DUOX, leading to HOCl signaling [11,46,49,52]. Both pathways are finalized by the generation of extracellular hydroxyl radicals that cause lipid peroxidation in the cell membrane, which then induces the mitochondrial pathway of apoptosis. Both pathways, initially act through extracellular RNS/ROS. However, the data presented in this study provide evidence that these pathways only have consequences for cell survival if an aquaporin-mediated influx of H_2_O_2_ into the cells is ensured in parallel. This is seen by the prevention of apoptosis induction after application of the aquaporin inhibitor Ag^+^. This aquaporin-mediated effect is strictly depending on the preceding inhibition of membrane-associated catalase. It seems to comprise a H_2_O_2_-dependent sensitization step for subsequent ROS/RNS signaling. This sensitization step seems to be completed within less than 1 h. As intracellular catalase is also inhibited by 3-AT, the intracellular resistance towards exogenous ROS/RNS signaling seems to be mediated by an enzyme system that is different from catalase. Glutathione peroxidase is the most likely candidate for this effect. The interdependency between intercellular ROS/RNS signaling and counteracting aquaporin action is reflected (i) in the lag phase of the apoptosis kinetics after catalase inhibition and (ii) in the restriction of the inhibitory effect of Ag^+^ exactly to this lag phase.

H_2_O_2_ intruding through aquaporins seemed to originate from the dismutation of NOX1-generated extracellular superoxide anions, as its effect was inhibited by the NOX1 inhibitor AEBSF and the catalase mimetic EUK-134. Importantly, H_2_O_2_ intruding through aquaporins was not the direct cause of apoptosis induction. Rather, it triggered a sensitizing step that allowed extracellular ROS/RNS signaling to become effective. The necessity for extracellular signaling is particularly demonstrated by strong and immediate inhibition of apoptosis induction through addition of the HOCl scavenger taurine even at late time points. If there had been a direct apoptosis-inducing effect of intruding H_2_O_2_, scavenging of HOCl by taurine would not have been sufficient to block of the apoptosis kinetics.

In total, these findings show that tumor cell-derived extracellular H_2_O_2_ has a function as substrate for extracellular HOCl synthesis, but also for the establishment of a second intracellular sensitizing effect. This sensitizing step seems to be mediated by aquaporins, as it is effectively prevented by inhibition of aquaporins. Both of these cooperating effects, HOCl signaling and aquaporin-mediated influx of H_2_O_2_, are efficiently controlled by membrane-associated catalase. Inhibition of catalase is therefore necessary to allow both of these interacting steps to become effective. NO/peroxynitrite signaling (that is initially not dependent on H_2_O_2_) also requires the sensitizing intracellular effect of H_2_O_2,_ mediated by aquaporins. The balance between aquaporin-mediated sensitizing effects of H_2_O_2_ that allow NO/peroxynitrite signaling, and H_2_O_2_-dependent interference with NO/peroxynitrite signaling is delicate. With increasing inhibition of catalase and resultant increase in free extracellular H_2_O_2_, NO/peroxynitrite signaling is suppressed on the expense of reactivated HOCl signaling. This suppression is mediated by complex iron-driven reactions [46].

The sensitizing effect of H_2_O_2_ that has passed the cell membrane through aquaporins can be completely mimicked through pretreatment of the tumor cells with (i) BSO, an inhibitor of glutathione synthetase or (ii) with sulfasalazine, an inhibitor of the xC transporter. Each one of these pretreatments leads to massive depletion of intracellular glutathione [43,64]. Pretreatment with BSO or sulfasalazine was not sufficient to induce apoptosis, thus confirming that an additional inducing step had to cooperate with this sensitizing step in order to achieve apoptosis induction. In our experiments, this inducing step was performed by intercellular ROS/RNS signaling. After catalase inhibition, apoptosis induction in the BSO- or sulfasalazine-pretreated cells by intercellular signaling no longer required the contribution of aquaporins. This was evident, as silver ions were no longer preventing apoptosis induction in this setting. This finding shows that the effect of H_2_O_2_ influx through aquaporins can be fully explained as well as substituted by the depletion of the intracellular glutathione level. In line with this conclusion, the kinetics of apoptosis induction in BSO- or sulfasalazine-pretreated tumor cells after addition of the catalase inhibitor started immediately, without showing the lag phase that is typical for control cells that had not been pretreated with BSO or sulfasalazine.

It is conceivable that glutathione depletion also allows inactivation of glutathione peroxidase through H_2_O_2_, leading to strong inhibition of the GSH/GPx system [65].

In glutathione-depleted cells, but not in control cells, very low concentrations of 3-AT led to apoptosis induction through the NO/peroxynitrite pathway. This finding demonstrates that very low concentrations of the catalase inhibitor were sufficient for reactivation of NO/peroxynitrite signaling, but that the parallel influx of H_2_O_2_ under these conditions was not sufficient to deplete glutathione to a level that would sensitize the cells for NO/peroxynitrite signaling. However, when GSH depletion was reached through BSO- or sulfasalazine pretreatment, the efficiency even of a low degree of catalase inhibition became overt. This interaction bears a chance for synergistic effects during tumor therapy, based on interactive glutathione depletion and catalase inhibition.

The sensitizing effect of lowering the glutathione level for the effects of extracellular ROS/RNS signaling can be best explained by the negative effect on the action of glutathione peroxidase 4. This enzyme uses glutathione to repair lipid oxidation in the membrane and thus has the potential to counteract the final step of intercellular RNS/ROS signaling [62]. Glutathione depletion prevents the performance of the catalytic cycle of glutathione peroxidase and may also have negative effects on the enzyme itself [65]. Glutathione depletion, either through intruding H_2_O_2_ or inhibition of glutathione synthesis through diminishment of the necessary building block cysteine or inhibition of glutathione synthase, seems to abrogate this negative interference with extracellular signaling.

**Figure 13:**
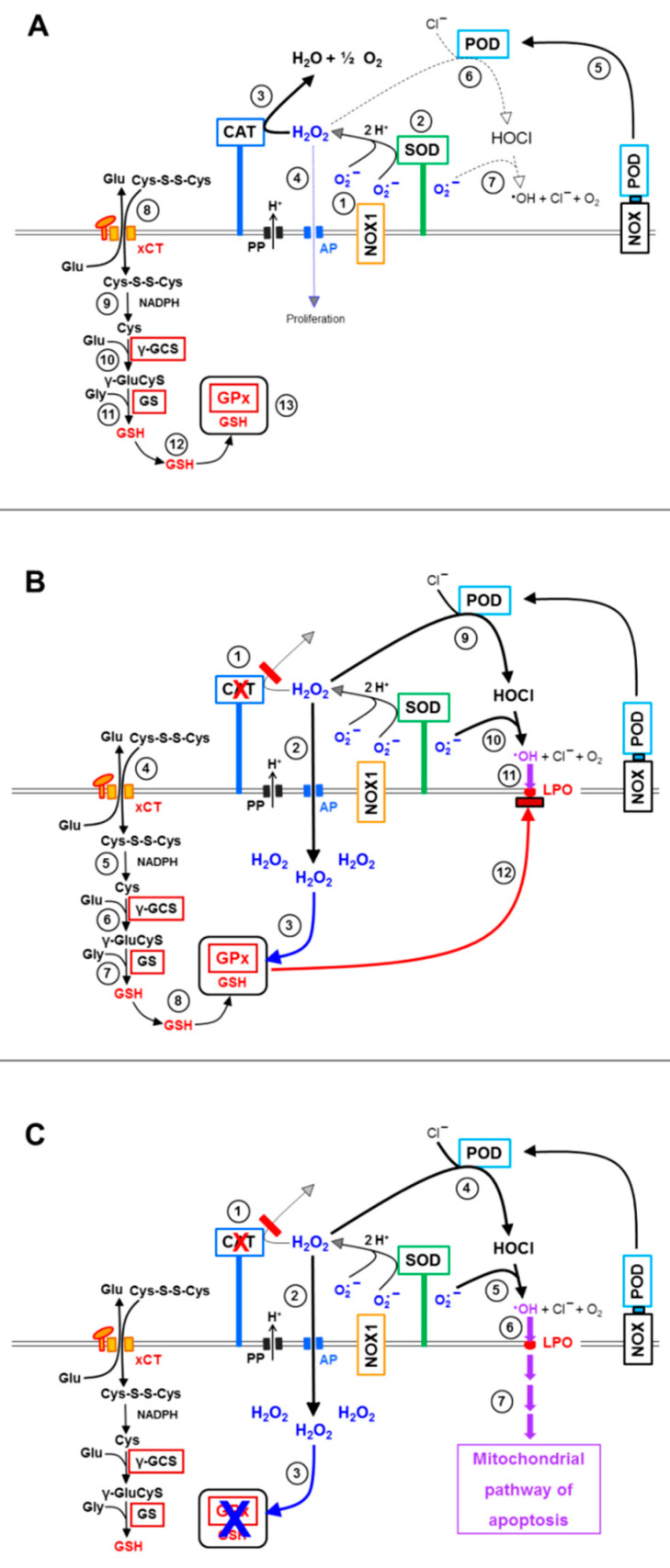
The interaction between membrane-associated catalase and aquaporins of tumor cells. (**A**). Catalase prevents HOCl signaling and influx of H_2_O_2_ through aquaporins. NOX1-derived superoxide anions (#1) are dismutated by membrane-associated SOD (#2) or spontaneously. The resultant H_2_O_2_ is decomposed by membrane-associated catalase (#3). A minor concentration of H_2_O_2_ may escape the action of immobilized catalase and enter the tumor cells through aquaporins (AP) (#4). This is required for proliferation stimulation. Membrane-associated dual oxidase (DUOX) releases its peroxidase domain (POD) through the action of matrix metalloproteases (#5). As long as catalase is acting, the potential generation of HOCl through H_2_O_2_/POD interaction (#6), as well as hydroxyl radical generation through the reaction between HOCl and superoxide anions (#7) do not take place. The xC transporter transports cystine into the cells for the exchange of glutamate (#9). Cystine is reduced to L-cysteine (#9). Gamma-glutamylcyseine synthase combines L-cysteine and L-glutamate (#10) to γ glutamylcysteine (γ-GluCys). Glutathione synthase then completes the formation of glutathione (GSH) (#11). Glutathione and Glutathione peroxidase (GPx) effectively decompose H_2_O_2_, peroxynitrite and repair lipid peroxides in the membranes. (**B**). Consequences of catalase inhibition. Inhibition of catalase (#1) allows the influx of H_2_O_2_ through aquaporins (#2). GPX/GSH decompose H_2_O_2_ (#3), gradually leading to consumption of GSH and inhibition of GPX. The action of the xC transporter and glutathione synthesis (#4–8) counteraction consumption of GSH. The second consequence of catalase inhibition is the reactivation of HOCl signaling (#9–11), which is finalized by hydroxyl radical-mediated lipid peroxidation (LPO). As long as GPx/GSH are functional, they counteract lipid peroxidation (#12) and in this way prevent apoptosis induction. (**C**). Final steps. As soon as influx of H_2_O_2_ through aquaporins has depleted glutathione and inactivated GPx (#2–3), HOCl signaling and lipid peroxidation (#4–6) are no longer counteracted by GPx/GSH and the mitochondrial pathway of apoptosis is induced (#7).

These findings allow the drawing of a hierarchical scheme of interactions between membrane-associated NOX1, catalase, aquaporins, xC transporters and intracellular glutathione peroxidase 4/glutathione (Figure 13).

**Figure 14:**
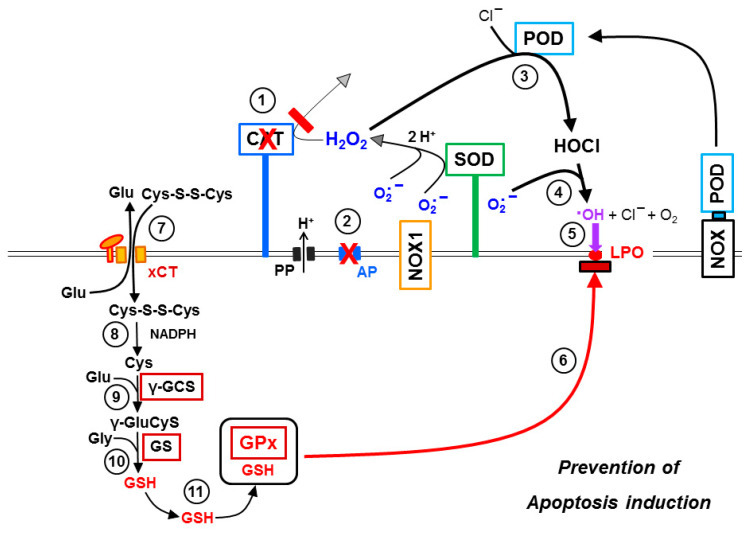
Consequences of parallel inhibition of catalase and aquaporins. When catalase (#1) and aquaporins (#2) are inhibited in parallel, apoptosis induction is prevented despite activated HOCl signaling (#3–5), as GPX/GSH actively interferes with the consequences of lipid peroxidation (#6). This interaction is sustained, as GPx/GSH is not attacked by intruding H_2_O_2_, due to the inhibition of aquaporins.

Membrane-associated catalase in cooperation with SOD protects the tumor cells (i) from intercellular HOCl signaling and (ii) from the aquaporin-mediated influx of NOX1/SOD-derived H_2_O_2_. In parallel, the xC transporter ensures optimal synthesis of intracellular glutathione (Figure 13A). Inhibition of membrane-associated catalase (Figure 13B) abrogates the decomposition of H_2_O_2_ and, therefore, leads to the onset of HOCl signaling, as well as to a strong aquaporin-mediated influx of H_2_O_2_ into the tumor cells. Intruding H_2_O_2_ counteracts the combined action of GSH/GPx towards the apoptosis inducing effects of lipid peroxidation resulting from HOCl signaling. The length of time where GSH/GPx remains effective is determined by the expression and activity of the xC transporter and by the efficiency of GSH synthesis. It is counteracted by expression and efficiency of aquaporins, as well as the availability of intruding H_2_O_2_. As soon as glutathione has been depleted (and potentially GPx at least partially inactivated), lipid peroxidation is no longer counteracted by glutathione peroxidase/glutathione and triggers the onset of the mitochondrial pathway of apoptosis (Figure 13C). The mutual dominant relationship between membrane-associated catalase and aquaporins, as worked out in this manuscript, is illustrated in Figure 14. It is demonstrated that parallel inhibition of catalase and aquaporins efficiently prevents apoptosis induction despite reactivation of HOCl signaling through inhibition of catalase.

**Figure 15:**
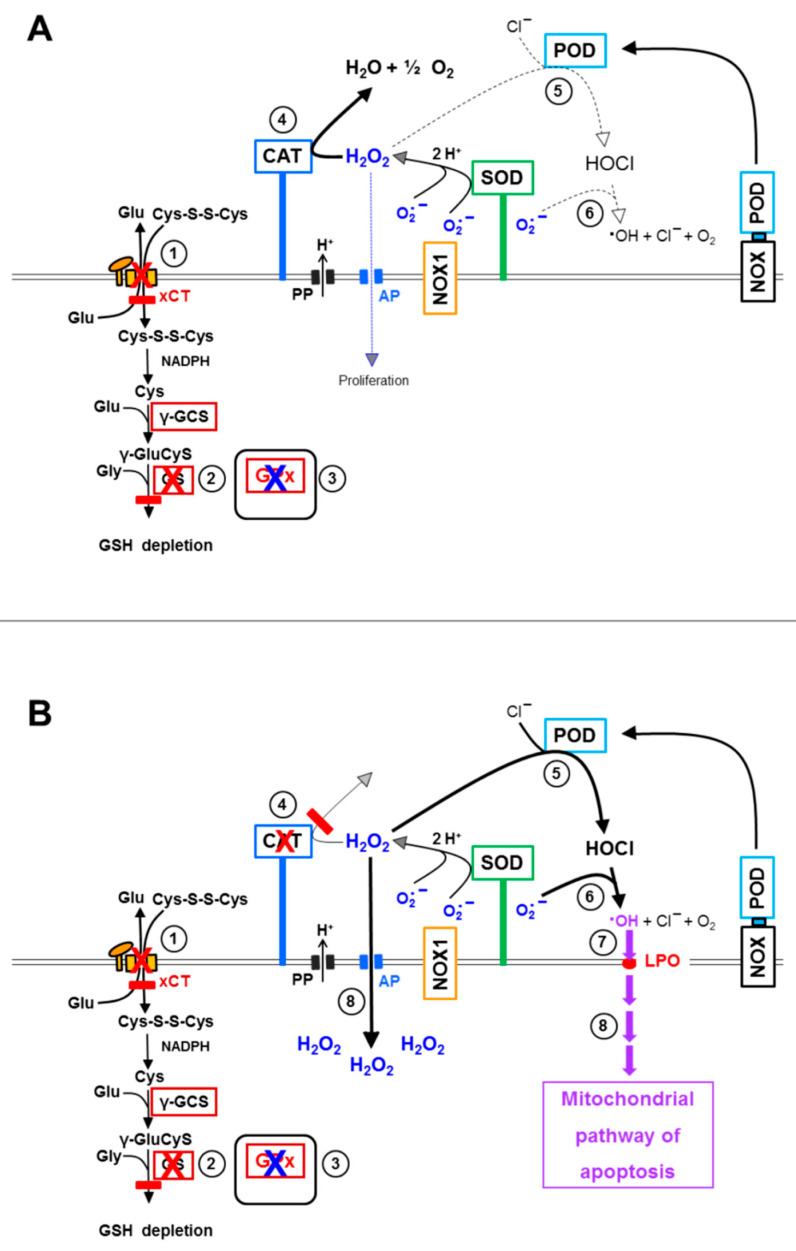
Consequences of inhibition of the xC transporter or glutathione synthase. (**A**): Prolonged inhibition of the xC transporter (#1) or of glutathione synthase (#2) lead to a lack of GSH and therefore nonfunctional GPx (#3). This has no impact on tumor cell survival, as long as membrane-associated catalase (#4) prevents HOCl signaling. (**B**): After preceding glutathione depletion through either inhibition of the xC transporter (#1) or of glutathione synthase (#2), GPx is nonfunctional (#3). Therefore, inhibition of catalase (#4) leads to immediate HOCl signaling (#5–7) and apoptosis induction (#8) without any lag phase.

The outstanding role of the GSH/GPx system is summarized in Figure 15. Prevention of GSH synthesis either through inhibition of the xC transporter or inhibition of glutathione synthase renders the effects that follow catalase inhibition independent of the action of aquaporins (Figure 15B). This is demonstrated through the omission of the lag phase in apoptosis induction when inhibition of GSH synthesis had been induced prior to catalase inhibition, as well as through the lack of inhibition by Ag^+^ under these conditions. Conversely, this interplay allows the conclusion that strong overexpression of xC transporters or glutathione synthase might prolong the lag phase between catalase inhibition and onset of apoptosis. Eventually this might even lead to resistance towards apoptosis induction by exogenous ROS/RNS attack. The recent findings by Bekuschus et al. [44] might possibly be explained by this mechanism.

**Figure 16:**
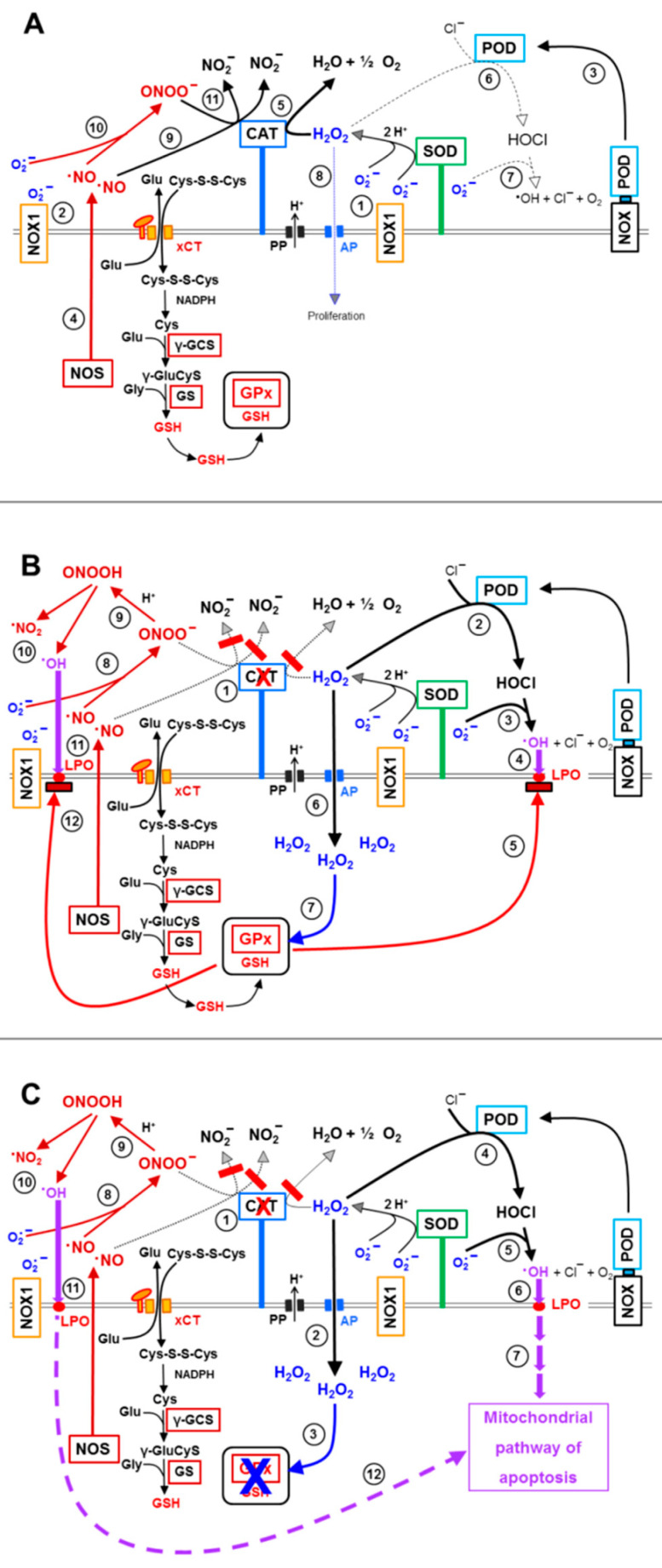
NO/peroxynitrite and HOCl signal interact with aquaporins. (**A**): Catalase protects towards ROS/RNS signaling. Superoxide anions generated by NOX1 are the basis for HOCl signaling (#1), as they lead to the formation of H_2_O_2_, and also for NO/peroxynitrite signaling (#2), as they are reaction partners for NO. HOCl signaling requires the presence of POD (#3), whereas NO/peroxynitrite signaling is determined by NOS-derived NO (#4). Membrane-associated catalase prevents HOCl signaling through decomposition of H_2_O_2_ (#5–7). As a consequence of H_2_O_2_ decomposition, the influx of H_2_O_2_ through aquaporins is minimal (#8). Catalase also prevents NO/peroxynitrite signaling, as it oxidizes NO (#9) in a two-step reaction that requires compound I of catalase, and as it decomposes peroxnitrite (#10). (**B**): Consequences of catalase inhibition. The inhibition of catalase (#1) reactivates HOCl signaling (#2–4), but the resultant lipid peroxidation (LPO) first remains without effects as it is efficiently repaired by GSH/GPx (#5), as long as the influx of H_2_O_2_ through aquaporins (#6, 7) has not achieved glutathione depletion. Inhibition of catalase abrogates oxidation of NO and decomposition of peroxynitrite and therefore reactivates NO/peroxynitrite signaling (#8–11). The resultant lipid peroxidation is blocked by GSH/GPx as well (#12). (**C**) Final steps. As a consequence of catalase inhibition (#1), H_2_O_2_ influx through aquaporins (#2) finally leads to the depletion of GSH (#3) and renders the complex GPx/GSH nonfunctional. Therefore, HOCl signaling (#4–6) and NO/peroxynitrite signaling (#8–11) are no longer counteracted by GPX/GSG and induce the mitochondrial pathway of apoptosis (#7, #10). Importantly, NO/peroxynitrite signaling and HOCl signaling act sequentially, but not in concert, due to complex consumption reactions between NO and H_2_O_2_ [63].

For simplicity, so far, the discussion had been focused solely on reactivation of HOCl signaling after inhibition of membrane-associated catalase. However, the situation in reality is more complex, as catalase acts as a multifunctional enzyme (Figure 16A). In addition to its classical function, i e., decomposition of H_2_O_2_, catalase can also oxidize NO and decompose peroxynitrite [30,66,67,68]. Inhibition of catalase, therefore, can also reactivate NO/peroxynitrite signaling (Figure 16B). As NO/peroxynitrite signaling is also finalized by lipid peroxidation through hydroxyl radicals, the counteraction of GSH/GPx towards the NO/peroxynitrite signaling pathway is analogous to that shown before for HOCl signaling. Thus, also NO/peroxynitrite signaling is only effectively induces apoptosis if aquaporin-mediated influx of H_2_O_2_ has caused an abrogation of the protective effect of GPx/GSH.

The dependency of apoptosis induction by intercellular RNS/ROS signaling on a dominant cooperative action of aquaporins was not only shown after direct inhibition of catalase by 3-AT, but also after treatment of compounds that cause catalase inactivation through different regimes. These were direct singlet oxygen generated by an illuminated photosensitizer, as well as modulation of cell-derived NO concentrations through the NOD inhibitors cyanidin and taxol, or the arginase inhibitor NOR-NOHA. Modulation of the NO level has been shown to lead to singlet oxygen generation [50]. Singlet oxygen has the potential to inactivate membrane-associated catalase and to trigger the generation of secondary singlet oxygen by targeted tumor cells [47,50,63]. Therefore, it may be concluded that mechanisms of tumor therapy that target membrane-associated catalase on NOX1-expressing tumor cells should require the cooperation of aquaporins with intercellular ROS/RNS-mediated signaling. This consideration also extends to the action of physical plasma and plasma-activated medium, which cause selective apoptosis induction in tumor cells in a singlet oxygen-mediated process [38,39,40,41]. Our study defines the hierarchy of membrane-associated catalase inactivation, reactivation of intercellular ROS/RNS-driven signaling, cooperating aquaporin function and counteraction by glutathione/glutathione peroxidase. As these interconnections have been established in MKN-45 tumor cells, which are the best studied tumor cells in our hands, it seems relatively easy to apply this knowledge to other tumor cells. It is predictable that varying degrees of NOX1, catalase, aquaporin, xCT, glutathione synthase and glutathione peroxidase expression can be expected in different tumor cell lines. This will most likely cause differences in the kinetic modes of the resultant interactions and subsequent degrees of apoptosis induction, as well as differential requirements to interfere with or enhance defined intermediate steps. Based on the established reaction scheme, a rational approach to handle these differences seems to be possible.

It is also obvious that the hierarchy of interactions established for MKN-45 cells is specifically relevant for tumor cells and has no impact on nonmalignant cells, as these lack the key features of active NOX1 and membrane-associated catalase.

## 5. Conclusions

Inactivation of membrane-associated catalase of tumor cells reactivates extracellular NOX1-driven ROS/RNS signaling through the NO/peroxynitrite or the HOCl signaling pathway. The apoptosis-inducing effect of extracellular ROS/RNS signaling is counteracted by intracellular glutathione/glutathione peroxidase. Apoptosis induction, therefore, requires abrogation of intracellular glutathione/glutathione peroxidase action through an influx of extracellular, tumor cell-derived H_2_O_2_ through aquaporins. ROS/RNS signaling, as well as aquaporin action, are under the control of membrane-associated catalase.

## Data Availability

All data obtained in this study are presented in the manuscript and its Supplementary Materials.

## Supplementary Materials

The following are available online at www.mdpi.com/article/10.3390/antiox10101585/s1, Figure S1: ROS/RNS signaling of tumor cells after catalase inhibition. Figure S2: Site of action of inhibitors and scavengers. Figure S3: Image of intact MKN-45 cells (A) and a mixture of intact and apoptotic MKN-45 cells (B) as seen through the inverted phase contrast microscope at high magnification. Figure S4: Demonstration of nuclear condensation/fragmentation through staining with bisbenzimide. Figure S5: Intact and apoptotic BG-1 ovarial carcinoma cells after staining with bisbenzimide. Figure S6: Apoptotic 208 F src3 cells with condensed/fragmented nuclei as determined by bisbenzimide staining/inverted fluorescence microscopy (A) show a positive TUNEL reaction, indicative of DNA strand breaks that are characteristic of apoptosis (B). Figure S7: Redox biology at the membrane of bona fide tumor cells. Figure S8: Redox biology at the membrane of transformed cells, i.e., cells from early stages of oncogenesis or transformed in vitro without passage in animals. Figure S9: Redox biology on the surface of non-malignant cells in the absence or presence of H_2_O_2_ or peroxynitrite.
