# Supplementary material for: Inhibition of Membrane-Associated Catalase, Extracellular ROS/RNS Signaling and Aquaporin/H2O2-Mediated Intracellular Glutathione Depletion Cooperate during Apoptosis Induction in the Human Gastric Carcinoma Cell Line MKN-45
Source: Antioxidants (Basel). 2021 Oct 9;10(10):1585. doi: 10.3390/antiox10101585 (PMC8533628; doi:10.3390/antiox10101585)
Supplement: Supplementary file 1 [file antioxidants-10-01585-s001.zip › antioxidants-1381193-supplementary.pdf]

Supplementary Materials

Inhibition of membrane-associated catalase, extracellular ROS/RNS signaling and aquaporin/H_2O_2-mediated intracellular glutathione depletion cooperate during apoptosis induction in the human gastric carcinoma cell line MKN-45

Georg Bauer [1, 2]

1) Institute of Virology, Medical Center - University of Freiburg

2) Faculty of Medicine, University of Freiburg, Freiburg, Germany

Contents of Supplementary Materials:

Supplementary Materials provides additional information about the following aspects related to the cooperation between RNS/ROS-dependent apoptosis-inducing signaling and aquaporin/H_2O_2-mediated glutathione depletion:

1. ROS/RNS-dependent apoptosis induction in tumor cells compared to nonmalignant cells.
2. The significance of inhibitors and scavengers used in this study
    2.1 The significance of inhibitor/scavenger studies for the elucidation of ROS/RNS signaling.
    2.2 Specific inhibitors/scavengers applied in this study.
3. The morphological characteristics of apoptotic tumor cells.
4. Supplementary discussion
5. Supplementary References

1. ROS/RNS-dependent apoptosis induction in tumor cells compared to nonmalignant cells.

This study is focusing on the control of tumor cell apoptosis induced by inhibition of membrane-associated catalase, which represents a hallmark of bona fide tumor cells, which are established at a late stage of oncogenesis [1-5]. Membrane-associated catalase explains the mechanism that is underlying the classical findings by Deichman and colleagues on the acquisition of a H_2O_2-catabolizing phenotype during tumorigenesis [6-10]. Bona fide tumor cells express membrane-associated NOX1, which generates extracellular superoxide anions [1-3,11, 12]. These are required to establish an autocrine proliferation stimulus, but also are the basis for two potential apoptosis-inducing signaling pathways. These are the HOCl and the NO/peroxynitrite signaling pathway [13-16]. The presence of membrane-associated catalase prevents apoptosis-inducing signaling through decomposition of H_2O_2 (thus preventing HOCl synthesis), oxidation of NO and decomposition of peroxynitrite (thus preventing NO/peroxynitrite signaling) [4]. NOX expression, followed by the generation of extracellular superoxide anions and their dismutation to H_2O_2 have a positive controlling function for the expression of membrane-associated catalase [5]. Protective catalase of tumor cells is covalently bound to the cell membrane or extracellular matrix through the action of transglutaminase [5]. Inhibition or inactivation of membrane-associated catalase, as well as its siRNA-mediated knockdown or prevention of the transglutaminase reaction are potential principles for novel approaches to utilize tumor cell redox biology for therapeutic application [17-21]. The present study significantly extends our previous mechanistic concepts by including the dominant controlling role of intracellular glutathione and glutathione peroxidase that counteract the apoptosis-inducing effect of lipid peroxidation which is

induced by hydroxyl radicals. These hydroxyl radicals are generated in close vicinity to the cell membrane by HOCl- as well as NO/peroxynitrite signaling.

The apoptosis-preventing role of glutathione peroxidase/glutathione is dependent on glutathione synthase, is enhanced by the xC transporter that positively affects glutathione synthesis, and is diminished by H_2O_2-mediated influx through aquaporins. A dominant controlling role of aquaporins has been proposed by Keidar and colleagues [22, 23] for induction of tumor cell apoptosis by cold atmospheric plasma or plasma-activated media, but has not been originally connected to the control of intercellular ROS/RNS signaling of tumor cells after inhibition of their protective catalase. The study presented in this manuscript has been triggered by the findings and concepts of Keidar, Yan and colleagues. The connection of their concepts with the concept catalase-controlled intercellular ROS/RNS signaling will be hopefully instrumental for further approaches to establish ROS/RNS-based strategies for tumor treatment.

The basic concept of the redox-relevant tumor cell phenotype described by NOX1 expression in connection with membrane-associated catalase has been experimentally confirmed *in vitro* so far for a multitude of human tumor cell systems derived from different organs. These are glioblastoma, astrocytoma, retinoblastoma, thyroid carcinoma and sarcoma, mammary carcinoma, small and large cell lung carcinoma, kidney carcinoma, gastric carcinoma, melanoma, liver adenocarcinoma, pancreatic carcinoma, colon carcinoma, bladder carcinoma, prostate carcinoma, ovarial carcinoma, cervical carcinoma, osteogenic sarcoma, fibrosarcoma, rhabdomyosarcoma, Ewing sarcoma, chondrosarcoma, neuroblastoma, Non Hodgkin lymphoma, T-Zell-leukemia, EBV-positive und –negative Burkitt-Lymphoma, and myeloma. This indicates that NOX-1 expression and balanced control of NOX-1-

dependent apoptosis-inducing signaling pathways seems to represent a rather general principle of tumor cell redox biology.

Nonmalignant cells are not expressing membrane-associated NOX1 and therefore do not generate extracellular superoxide anions [1, 2, 11, 12, 15, 16, 24, 25, 26]. This can be convincingly shown by their nonresponsiveness to the treatment with NO donors or HOCl in the µmolar concentration range [15, 16, 26]. This non-responsiveness is explained by the lack of extracellular superoxide anions which are necessary for the following central reactions relevant for lipid peroxidation and apoptosis induction:

A. NO/peroxynitrite signaling pathway:

a) $^{\bullet}NO + \mathbf{O_2^{\bullet -}} \rightarrow ONOO^-$

b) $ONOO^- + H^+ \rightarrow ONOOH$

c) $ONOOH \rightarrow {}^{\bullet}NO_2 + \mathbf{{}^{\bullet}OH}$

B. HOCl signaling pathway

d) $HOCl + \mathbf{O_2^{\bullet -}} \rightarrow \mathbf{{}^{\bullet}OH} + Cl^- + O_2$

Therefore, nonmalignant cells cannot establish autocrine apoptosis-inducing signaling through the HOCl and NO/peroxynitrite signaling pathway, as they cannot be the target for the superoxide anion-dependent ROS/RNS effects. However, the release of Duox-coded peroxidase from nonmalignant cells can act in trans on malignant cells from early stages of oncogenesis [27]. As the expression of membrane-associated catalase is positively controlled by active NOX1 [5], nonmalignant cells do not express membrane-associated catalase. This has been confirmed by immunostaining [4]. *In line with these findings, the treatment of*

nonmalignant cells with catalase inhibitors such as 3-AT, neutralizing antibodies towards catalase, catalase-inactivating singlet oxygen or siRNA-mediated knockdown of catalase do not cause an apoptosis-inducing effect [4, 24, 28, 20, 29]. As it has been repeatedly established that the treatment modalities used in this study for apoptosis induction in tumor cells, have no apoptosis-inducing effect on nonmalignant cells, these cells have not been included into this study that is focusing on the redox-related chemical biology of tumor cells.

However, it is known that ROS/RNS-mediated apoptosis induction in nonmalignant cells can be achieved i) through the application of exogenous H_2O_2 which enters the cells through aquaporins [30, 31] or ii) through application of exogenous, preformed peroxynitrite [16]. H_2O_2-dependent apoptosis-induction in nonmalignant cells seems to be mediated by intracellular Fenton chemistry [32]. These aspects are further discussed in chapter 4 of this Supplement.

2. The significance of inhibitors and scavengers used in this study

2.1 The significance of inhibitor/scavenger studies for the elucidation of ROS/RNS signaling.

Intercellular ROS/RNS-mediated apoptosis induction through HOCl and NO/peroxynitrite signaling is not determined by the interactions of reaction partners that are in a uniform distribution. It rather represents a complicated example of surface chemical biology. It is determined by elements that are immobilized to the membrane of malignant cells, such as NOX-1, catalase, SOD, aquaporins and proton pumps. These elements are interacting with elements that are inside the cells, like NO synthase (NOS) that releases NO that may pass the membrane, and elements

like the peroxidase domain of DUOX that are initially bound to the membrane, but then are released through the action of matrix metalloproteases [13, 33]. Together, these elements initiate a multitude of biochemical interactions driven by short- and long-lived (i. e. highly and less reactive), nonradical and radical, damaging and non-damaging members of the ROS/RNS family. The mere description or even precise quantitation of these ROS/RNS per se cannot lead to solid conclusions in most cases, as the biological effects are determined by site-specific reactions. However, the use of inhibitors or scavengers can determine the causal role of defined species in signaling. The analytic principle thereby is to determine whether specific removal of a compound abrogates apoptosis induction. The apparent discrepancy between this approach, based on interference, with precise determination and quantitation of compounds can be easily explained.

A) When preformed HOCl is added to malignant cells, the interaction between HOCl and NOX-1-derived extracellular superoxide anions leads to the formation of hydroxyl radicals in close vicinity to the cell membrane. This causes apoptosis-inducing lipid peroxidation [34]. The site-specificity of this reaction is determined by the relatively low free diffusion path length of superoxide anions and the extremely short free diffusion path length of hydroxyl radicals [13, 15, 35]. Apoptosis induction in this experimental system can be prevented by scavenging HOCl by taurine, by preventing superoxide anion generation by AEBSF-mediated inhibition of NOX-1, by scavenging superoxide anions by SOD and by scavenging hydroxyl radicals by mannitol, dimethyl urea or terephthalate. B) Addition of 20 μM ferrous iron together with HOCl leads to efficient Fenton-like reaction of HOCl

($HOCl + Fe^{++} \rightarrow {}^{\bullet}\mathbf{OH} + Cl^{-} + Fe^{+++}$).

As HOCl is a far-ranging molecular species, and as the majority of HOCl is distant of the cell membrane at the moment of addition of ferrous iron, the majority of the

hydroxyl radicals will be generated at a distance larger than 40 nm from the cell membrane and therefore have no damaging effect on the membrane. The result, as determined by experiment, is the lack of apoptosis induction by HOCl in the presence of ferrous iron [34].

If the analysis would have been founded on the quantitation of hydroxyl radicals, both experimental situations as described under A and B would have led to a measurement of hydroxyl radicals, It is very likely that under the conditions of B the overall concentration of hydroxyl radicals would even been much higher than under A, where hydroxyl radical formation was restricted to the site of the membrane. Therefore, the determined association of hydroxyl radical generation with a lack of HOCl-mediated apoptosis induction as well as with successful HOCl-mediated apoptosis induction would not have allowed to conclude on a role of hydroxyl radicals in apoptosis induction – just by application of formal logics. However, the functional analysis by inhibitors allowed to reconstruct the chemical interactions.

A second example is based on the chemical biology of peroxynitrite. Experts in the field know that the reaction between peroxynitrite and CO_2, leading to nitrosoperoxycarboxylate ($ONOOCOO^-$), that decomposes into carbonate radicals and $^\bullet NO_2$, is 100 fold more efficient than the protonation of peroxynitrite and subsequent hydroxyl radical generation [36]. Therefore, hydroxyl radical generation through protonation of peroxynitrite and its decomposition was frequently denied in scientific discussions and carbonate radicals were regarded as more likely candidate for damaging effects. However, functional interference with scavengers and inhibitors showed that peroxynitrite-dependent apoptosis induction was prevented by FeTPPS, a decomposition catalyst for peroxynitrite and by hydroxyl radical scavengers, whereas scavengers of carbonate radicals had no inhibitory effect [17]. In addition, the inhibition of proton pumps by omeprazole caused complete inhibition of

peroxynitrite-mediated apoptosis induction. Therefore, the inhibitor data favoured the reaction of peroxynitrite that seemed not to be in line with the established chemistry of this compound. The resolution of this discrepancy is possible by addressing the site specific effects. There is no doubt that under conditions of equal distribution, the reaction between CO_2 and peroxynitrite prevailed and protonation of peroxynitrite was a background phenomenon. However, close to the membrane and its proton pumps, protonation of peroxynitrite seemed to be effective and responsible for the biological effect. Without doubt, the reaction between CO_2 and peroxynitrite will occur efficiently distant of the membrane, with the sole effect that it reduces the available concentration of peroxynitrite for protonation. Assuming the analysis would not have been based on inhibitors, but rather on the measurement of radical species, the high concentration of carbonate radicals in the medium compared to the conceivably lower concentration of hydroxyl radicals (due to the very limited range of reaction) would probably have been taken as an indication for the role of carbonate radical formation for the induction of apoptosis, and thus would have missed the correct conclusion.

2.2 Specific inhibitors/scavengers applied in this study

Supplementary Figure S1 summarizes NOX-1-driven apoptosis-inducing signaling through the HOCl and the NO/peroxynitrite signaling pathways under conditions of inhibition of membrane-associated catalase. This Figure is identical to Figure 16 C in the main manuscript. Its purpose is to allow the reader to easily overview the signaling components and their reactions. This is the basis for a an easy access to get acquainted with the sites of action of specific inhibitors/scavengers that have been used in this study and that are presented in Supplementary Figure S2.

Supplementary Figure S1

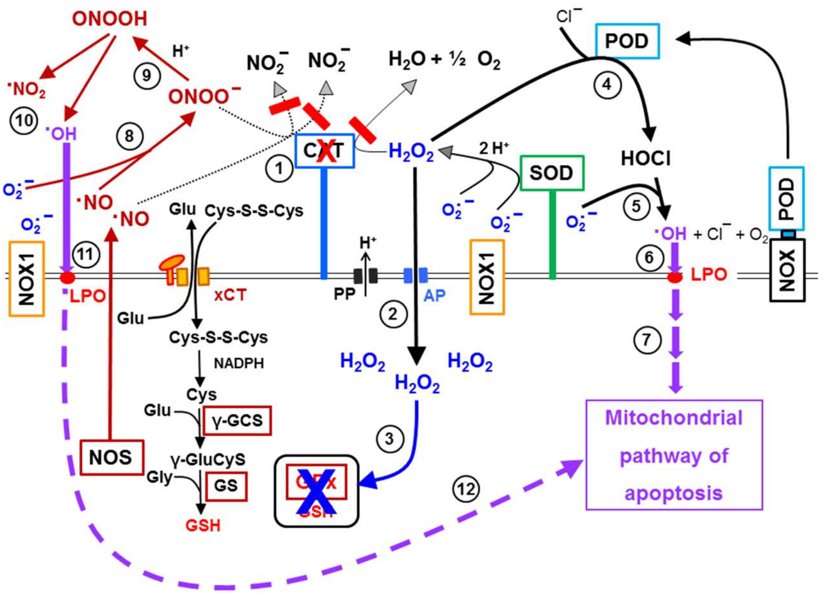

Supplementary Figure S1. **ROS/RNS signaling of tumor cells after catalase inhibition**. As a consequence of catalase inhibition (#1), H_2O_2 influx through aquaporins (#2) finally leads to the depletion of GSH (#3) and renders the complex GPx/GSH non-functional. Therefore, HOCl signaling (#4-6) and NO/peroxynitrite signaling (#8-11) are no longer counteracted by GPX/GSG and induce the mitochondrial pathway of apoptosis (#7, #12). Importantly, NO/peroxynitrite signaling and HOCl signaling act sequentially, but not in concert, due to complex consumption reactions between NO and H_2O_2 [14]. This figure is identical to Figure 16 C in the main manuscript.

Supplementary Figure S2

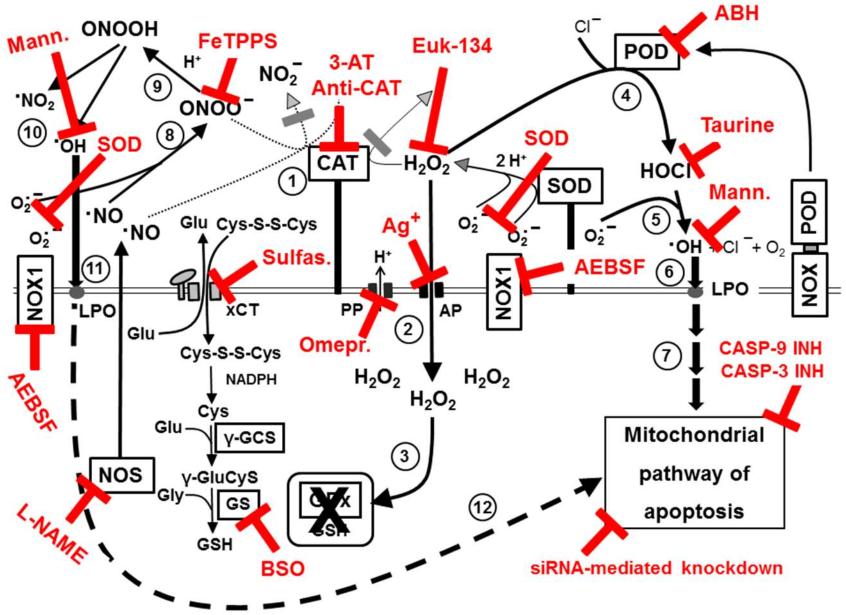

Supplementary Figure S2. Site of action of inhibitors and scavengers.
Please find explanation in the text.
Omepr. = omeprazole; CASP-9 INH = Caspase-9 inhibitor; CASP-3 INH = Caspase-3 inhibitor; siRNA = small interfering RNA; BSO = buthionine sulfoximine; Sulfas. = sulfasalazine; mann = mannitol; Anti-CAT = neutralizing antibody directed towards catalase;

Detailed description of inhibitors:

AEBSF (4-(2-Aminoethyl)benzenesulfonyl fluoride) has been described as an efficient inhibitor for NOX1 [37]. We have confirmed the efficiency of this inhibitor through demonstrating its strong inhibitory effect on superoxide anion-dependent formation of peroxynitrite after reaction with NO, and by demonstrating the inhibition of hydroxyl radical formation through superoxide anion / HOCl interaction by AEBSF [4, 13, 14, 34]. The inhibitory effect of increasing concentrations of AEBSF has been experimentally determined through titration of residual superoxide anion concentrations with SOD [Bauer, unpublished], following the protocol by [38]. It was found that 100 µM AEBSF caused 95 percent, 33 µM AEBSF caused 88 percent, and 11 µM AEBSF caused 75 percent inhibition of superoxide anion generation. AEBSF inhibits the generation of superoxide anions and therefore also prevents the formation of their dismutation product H_2O_2.

SOD (superoxide dismutase) is a classical scavenger of superoxide anions. The efficient inhibition of superoxide anion-dependent processes has been confirmed by us in more than thousand experiments during the last 20 years [13, 14, 15, 16, 26] As Cu,Zn-SOD shows a bell-shaped inhibition curve [see supplement of reference #39 for details], its application requires the knowledge on the range of superoxide anion concentration in the experimental system. If Cu/Zn-SOD is applied in relative excess compared to superoxide anions, the enzymatic SOD cycle cannot be not completed. Then the $SODCu^+$ intermediate, resulting from the interaction between the first superoxide anion with the enzyme, acts as a “Fenton reagent” on HOCl, yielding apoptosis-inducing hydroxyl radicals. Alternatively, the $SODCu^+$ intermediate can interact with NO, leading to the formation of a nitroxyl anion (NO^-) . The reaction of NO^- with molecular oxygen then leads to peroxynitrite [39], establishing the right side of the bell-shaped curve. As Mn-SOD (derived from E. coli) does not show this

biphasic pattern of reaction, we prefer to use Mn-SOD in our inhibitor experiments, being rather sure of unproblematic use of sufficiently high concentrations of the enzyme.

SOD removes superoxide anions, but generates H_2O_2 through this process.

EUK-134 is a salen manganese complex [40, 41] with catalase-mimetic properties. It is cell-permeable and acts in a catalytic mode. We have studied the application of salen manganese complexes in details with respect to their use in the analysis of ROS/RNS signaling [42]. We confirmed that EUK-134 up to a concentration of 25 µM rather specifically decomposed H_2O_2 and therefore can be used as a “catalase mimetic” that is independent of classical catalase inhibitors. At much higher concentrations, the interaction of EUK-134 with HOCl as well as its potential to generate HOCl in the presence of limiting concentrations of H_2O_2 were prominent.

ABH (4-aminobenzoyl hydrazide) is a specific inhibitor of peroxidase and efficiently prevents HOCl synthesis by myeloperoxidase or the DUOX-coded peroxidase domain. Its efficiency has been determined in hundreds of own experiments [13, 15, 26]. The inhibitory effect of ABH is analogous to siRNA-mediated knockdown of DUOX1 [43], confirming its target specificity. The specificity of ABH is further strongly assured by its mode of action. ABH represents a mechanism-based inhibitor which first is transferred into its radical form specifically by peroxidase, before it inhibits the peroxidase [44-46].

Taurine is a specific scavenger of HOCl [47]. Its strong inhibitory effect on the HOCl pathway and lack of inhibition of NO/peroxynitrite signaling has been confirmed several hundred times in our experiments [4, 13, 43]. Addition of 50 mM taurine

completely prevented apoptosis induction up to a concentration of 125 µM HOCl. At 250 µM HOCl, its inhibitory effect was still 70 percent [34]. Therefore, taurine is an excellent scavenger for the easy discrimination between HOCl and NO/peroxynitrite signaling.

Mannitol is an established hydroxyl radical scavenger [48]. In line with its published potential, 20 mM mannitol completely prevented HOCl- as well as NO/peroxynitrite signaling after inhibition of membrane-associated catalase of tumor cells [4]. Both pathways are finalized by the action of hydroxyl radicals. It was also experimentally confirmed that the effect of mannitol was analogous to the effects determined by the hydroxyl radical scavengers dimethylthiourea, terephthalate and DMSO [20, 26, 32, 49]. The use of mannitol or dimethyl thiourea allows to differentiate between extracellular and intracellular hydroxyl radicals [32].

L-NAME is an arginine analogue prodrug and needs to be hydrolyzed *in vitro* and *in vivo* to the active inhibitor NG-nitro-L-arginine [50]. In the concentration used in our system (2.4 mM) it is inhibitory for all isoforms of NOS. We found L-NAME to be a reproducibly acting, specific inhibitor that did not interfere with HOCl signaling and inhibited NO-dependent processes based on NOS action, while it did not interfere with the effects of NO donors.

FeTPPS is a an iron porphyrin complex that decomposes peroxynitrite in an efficient catalytic mode [51, 52]. We found in several hundreds of experiments that FeTPPS did not interfere with HOCl signaling, but completely blocked apoptosis induction after superoxide anion/NO interaction. It was not interfering with NO-specific, peroxynitrite-independent processes like consumption of H_2O_2 by NO (Bauer in preparation). 25

µM FeTPPS were shown to completely block the apoptosis inducing effect of 100 µM presynthesized peroxynitrite [29], confirming the catalytic nature of its inhibitory action

Omeprazol is an established inhibitor of proton pumps. Its application prevented the protonation of peroxynitrite and subsequent generation of apoptosis-inducing hydroxyl radicals.

The specificity and efficiency of **caspase-9** and **caspase-3 inhibitors** has been confirmed by the producers (R&D Systems, Wiesbaden). These inhibitors completely blocked apoptosis induction through the mitochondrial pathway of apoptosis. The significance of these inhibitory effects was ascertained, as siRNA-mediated knockdown of catalase-9 or catalase-3 completely prevented apoptosis induction through HOCl- and NO/peroxynitrite signaling [43].

The role of the **mitochondrial pathway of apoptosis** was ensured through the inhibitory effect of siRNA-mediated knockdown of sphingomyelinase, voltage-dependent anion channel, Bak, Diablo, mitochondrial SOD, cytochrome c, APAF, caspase-9 and caspase-3 on apoptosis induction through the HOCl and the NO/peroxynitrite pathway [43].

The efficiency of **Ag^+** as aquaporin inhibitor [22, 23] was confirmed through functional analysis as described in Figure 1 of the main manuscript.

.

Sulfasalazine is an established inhibitor of the xC transporter [53].

Its efficiency was proven by the functional analysis described in the main manuscript, as pretreatment of tumor cells with sulfasalazine caused an analogous result as preventing glutathione synthesis.

BSO is an established inhibitor of glutathione synthase. Its efficiency was proven by functional assays. BSO treatment leads to a dramatic decrease of glutathione detectable by monochlorobimane staining [54]

3-aminotriazole (3-AT) is an established catalase inhibitor [55]. Its functional relevance for tumor cell apoptosis has been confirmed by functional assays [4], as its application resulted in reactivation of intercellular ROS/RNS signaling of tumor cells, depending on the catalase substrates H_2O_2, NO and peroxynitrite. Inactivation of membrane-associated catalase of tumor cells was directly determined through measuring the abrogation of resistance towards exogenous peroxynitrite [4, 5] in a specific assay for the activity of membrane-associated catalase [5, 29]. In addition, the additive effect of concentration-dependent siRNA-mediated catalase knockdown and application of 3-AT confirms its action directed towards the activity of catalase.

Inhibition of membrane-associated catalase by **neutralizing antibodies directed towards human catalase** or with the catalase inhibitor 3-aminotriazole (3-AT) was shown to reactivate intercellular ROS/RNS signaling, which requires the catalase substrates H_2O_2, NO and peroxynitrite (4, 18). In addition, a peroxynitrite challenge of tumor cells pretreated with catalase-neutralizing antibodies or with 3-AT directly confirmed catalase inhibition [4, 56, 29]. Challenge of tumor cells with exogenous peroxynitrite in the presence of AEBSF (for the prevention of inhibitory side reactions)

has been established as specific assay for the modulation of membrane-associated catalase of tumor cells [29].

The significance of the results obtained by the inhibitors used in this study and presented in this Supplement has also been confirmed by reconstitution experiments. In this experimental approach, elements that had been determined to be essential for a specific signaling step by inhibition experiments were used in reconstitution experiments that were counter-controlled by inhibitors. For example, after elucidation of the basic elements of the HOCl signaling pathway, HOCl was added to nonmalignant and malignant cells and the apoptotic response was inhibited by the analogous inhibitors that had been used for the elucidation of the pathway. After elucidation of the NO/peroxynitrite pathway through inhibitor studies, NO donors as well as chemically synthesized peroxynitrite were used in reconstitution experiments, counter-controlled by inhibitors. This approach allowed to define specific and reproducibly acting inhibitors/scavengers that were useful for further studies. Another principle of evaluation of the analytical quality of inhibitors was to target the same target with different inhibitors and determine whether the results were congruent. For example, it was shown that inhibition of NOX-1 by AEBSF or apocynine, scavenging of superoxide anions by Cu/ZnSOD, FeSOD or MnSOD, scavenging of superoxide anions by several synthetic SOD mimetics was causing the same inhibitory effect on superoxide anion-driven processes. Finally, the use of specific siRNAS allowed to determine whether knockdown of DUOX had the same effect as scavenging HOCl by taurine, and whether reconstitution of the tumor cells pretreated with siRNA towards DUOX with active myeloperoxidase would reestablish HOCl signaling, as determined by inhibition with taurine. In analogy, it was confirmed that siRNA-mediated knockdown of NOS had the same inhibitory effect on NO/peroxynitrite signaling (and

no effect on HOCl signaling). It was then determined, whether the effect of NOS knockdown could be abrogated by supplementing the system with NO from an NO donor.

3. The morphological characteristics of apoptotic tumor cells

Apoptotic MKN-45 cells were determined and quantified according to the classical criteria established by Kerr et al. and Elmore et al., i. e. nuclear condensation and fragmentation [57, 58]. Apoptotic MKN-45 cells can be clearly differentiated from intact MKN-45 cells by phase contrast microscopy, provided the inverted microscope has an excellent phase contrast equipment and is professionally adjusted. The observing and quantifying scientist requires profound training, experience and also disciplined endurance. Under these conditions, the use of phase contrast microscopy as analytical tool allows to quantify the percentage of apoptotic cells kinetically in the same assays and to perform differential addition of inhibitors etc. without disaggregating the cell system.

Supplementary Figure S3 illustrates the difference between intact MKN-45 cells and apoptotic MKN-45 cells with condensed and fragmented nuclei.

Supplementary Figure S3

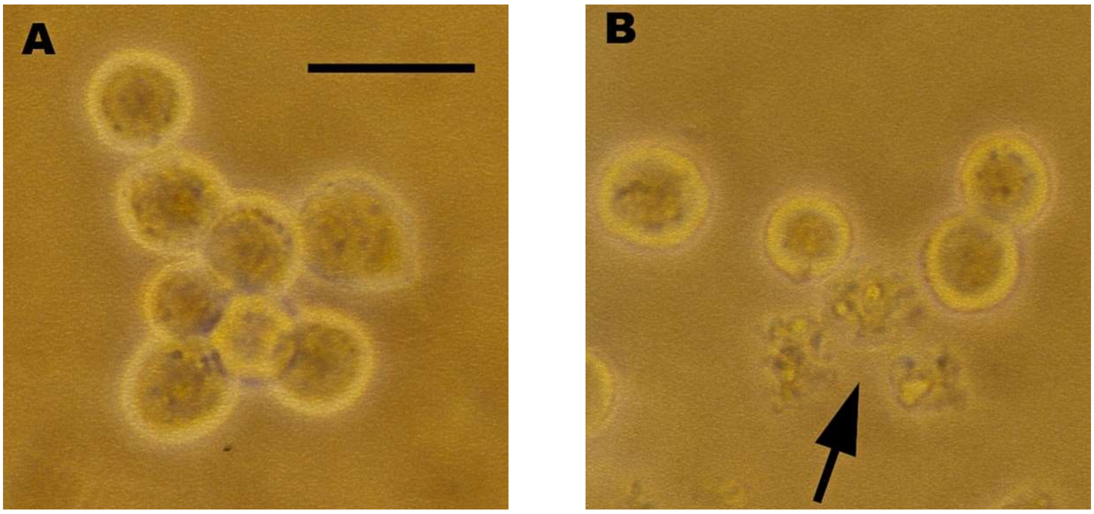

Supplementary Figure S3: Image of intact MKN-45 cells (A) and a mixture of intact and apoptotic MKN-45 cells (B) as seen through the inverted phase contrast microscope at high magnification. The arrow indicates the apoptotic cells that are characterized by nuclear condensation and fragmentation. The apoptic cell on the left also shows residual membrane-blebbing. The bar in A indicates 50 µm.

The difference between large, intact nuclei of intact cells and condensed/fragmented nuclei of apoptotic cells can be controlled by staining with the nuclear stain bisbenzimide as shown in Suppl. Figure S4.

Supplementary Figure S4

A B

C D

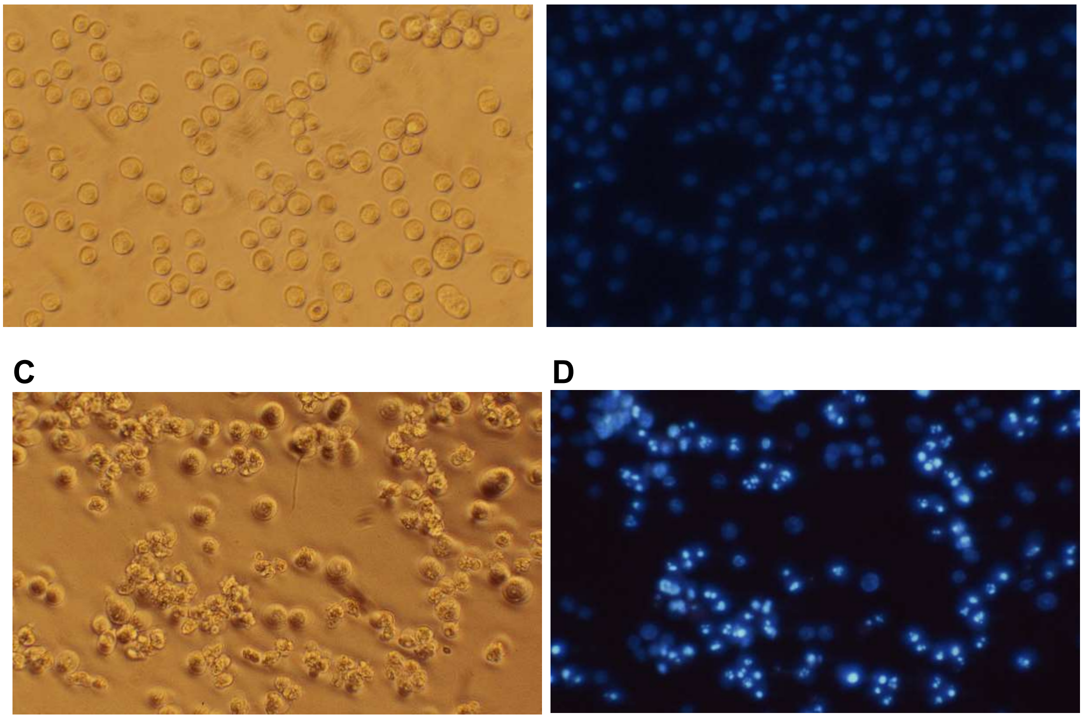

Supplementary Figure S4: Demonstration of nuclear condensation/fragmentation through staining with bisbenzimide. The figure shows intact MKN-45 cells (A, B) and

a population of MKN-45 cells with more than 50 % apoptotic cells after catalase inhibition (C, D). The cells in the pictures on the right had been stained with 1 µg/ml bisbenzimide for 40 min and were either inspected by inverted phase contrast microscopy (A, C) or by inverted fluorescence microscopy (B, D).

Staining with bisbenzimide can be successfully applied for apoptosis induction in many different tumor cell lines. Supplementary Figure S5 demonstrates the very low percentage of condensed/fragmented nuclei in intact ovary carcinoma cells BG-1 (left) and the high percentage of apoptotic cells with condensed/fragmented nuclei in BG-1 cells treated by catalase inactivation.

Supplementary Figure S5

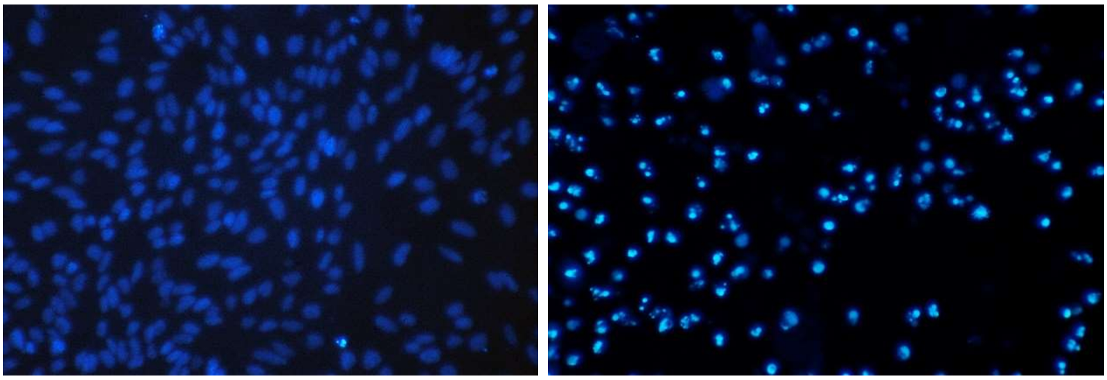

Supplementary Figure S5: Intact and apoptotic BG-1 ovarial carcinoma cells after staining with bisbenzimide.

Control assays during establishment of our analytical system ensured that morphological changes characterized as nuclear condensation/fragmentation correlate well with a positive TUNEL reaction (Terminal deoxynucleotidyl transferase dUTP nick end labeling reaction), which is indicative of DNA doublestrand breaks, as it is based on the addition of fluorescent dUTP to free 3`hydroxyl ends, mediated by terminal nucleotidyl transferase [16, 59].

Supplementary Figure S6 shows 208F src3 cells after autocrine apoptosis induction [60] show a high percentage of condensed/fragmented nuclei indicative for apoptosis

that can be determined by bisbenzimide staining / inverted fluorescence microscopy (A) and confirmed to exhibit DNA strand breaks that are visualized by TUNEL-positivity (B), whereas intact cells with large nuclei and low bisbenzimide staining are negative in the TUNEL reaction.

Supplementary Figure S6

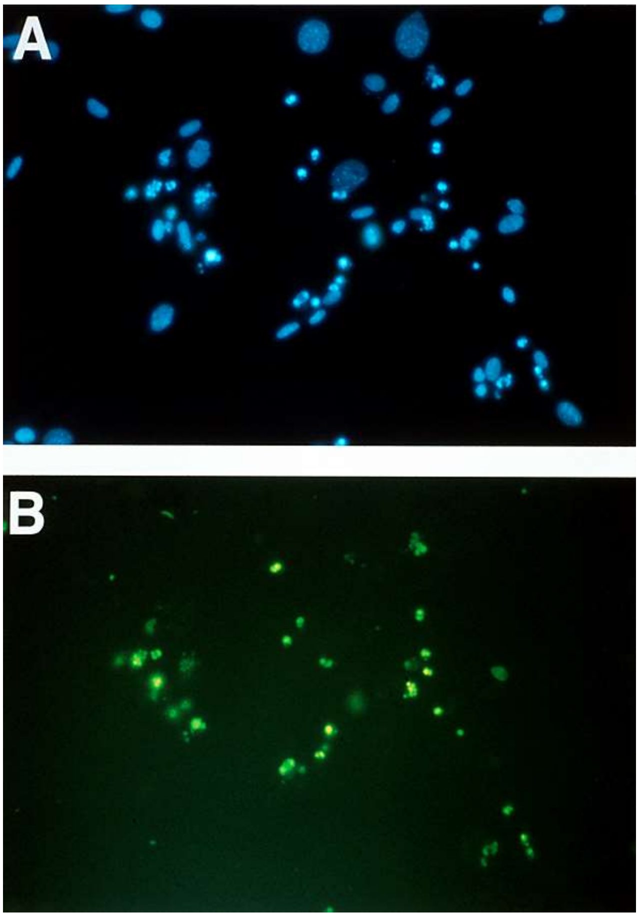

Supplementary Figure S6: Apoptotic 208 F src3 cells with condensed/fragmented nuclei as determined by bisbenzimide staining / inverted fluorescence microscopy (A) show a positive TUNEL reaction, indicative of DNA strand breaks that are characteristic of apoptosis (B). In contrast, intact cells with weak bisbenzimide staining and intact, large nuclei are not stained by the TUNEL reaction. The cells had undergone autocrine ROS-mediated apoptosis induction through the HOCl signaling pathway and were subjected to the TUNEL reaction and stained with bisbenzimide. The differentiation between the results of the two methods was achieved through the use of the appropriate filter systems.

Further controls ensured that positive annexin V staining, indicative of the exposure of phosphatidylserine to the outside of cells during the early phase of apoptosis preceded the detectability of condensed nuclei [56].

The unambiguous correlation between the morphological features determined in our experiments with characteristics of apoptotic cell death was also ascertained through the use of specific caspase-3 and caspase-9 inhibitors [4]. As these inhibitors completely blocked apoptosis induction mediated by the HOCl and the NO/peroxynitrite signaling pathway, caspase-dependent apoptosis was demonstrated. The strong inhibitory effect of caspase-9 inhibitor assured that the process was mediated by the mitochondrial pathway of apoptosis. This was further confirmed by siRNA-based knockdown of caspase-9 and caspase-3, as well as knockdown of specific elements relevant for the mitochondrial pathway of apoptosis, such as bak, VDAC, cytochrome C, APAF, mitochondrial SOD and Diablo [43]. Knockdown of each one of these elements caused strong inhibition of apoptosis induction by HOCl and NO/peroxynitrite signaling. The inhibitory effect of the siRNA-mediated knockdown of sphingomyelinase indicated the role of ceramides during execution of apoptosis.

4. Supplementary Discussion

The focus of this study is on selective apoptosis induction in tumor cells, with the aim to understand the interaction between essential control elements like membrane-associated catalase, NOX-1, aquaporins, glutathione peroxidase, glutathione synthase and xC transporter. Nevertheless, it is also challenging to discuss the redox-related chemical biology of transformed cells, i. e. malignant cells from early stages of oncogenesis as well as the redox biology of non-malignant cells.

This supplementary discussion is based on experimental findings from this study, but also takes advantage of the large body of knowledge established by colleagues working in this field world-wide and extends to some hypothetical considerations.

It seems useful to recapitulate the central conclusions from our study, summarized in Supplementary Figure S7, which is based on Figure 13 in the main manuscript. Supplementary Figure S7 A demonstrates that tumor cells prevent NOX-1-driven HOCl signaling as well as aquaporin-mediated influx of H_2O_2 through expression of membrane-associated catalase. The analogous control of NO/peroxynitrite signaling is not shown in the figure in order to reduce the complexity of the picture. Inhibition of membrane-associated catalase (Supplementary Figure S7 B) allows reactivation of HOCl signaling, with the consequence of lipid peroxidation through hydroxyl radicals. However, the apoptosis-inducing effect of lipid peroxidation is initially blocked through the action of glutathione peroxidase -4/glutathione. Intruding H_2O_2 leads to glutathione depletion and thus allows the full effect of lipid peroxidation on apoptosis induction through the mitochondrial pathway (Supplementary Figure S7 C). Based on its established signaling potentials, the lipid peroxidation product 4-HNE might also contribute to the inhibition of glutathione peroxidase, to glutathione depletion and to

Supplementary Figure S7

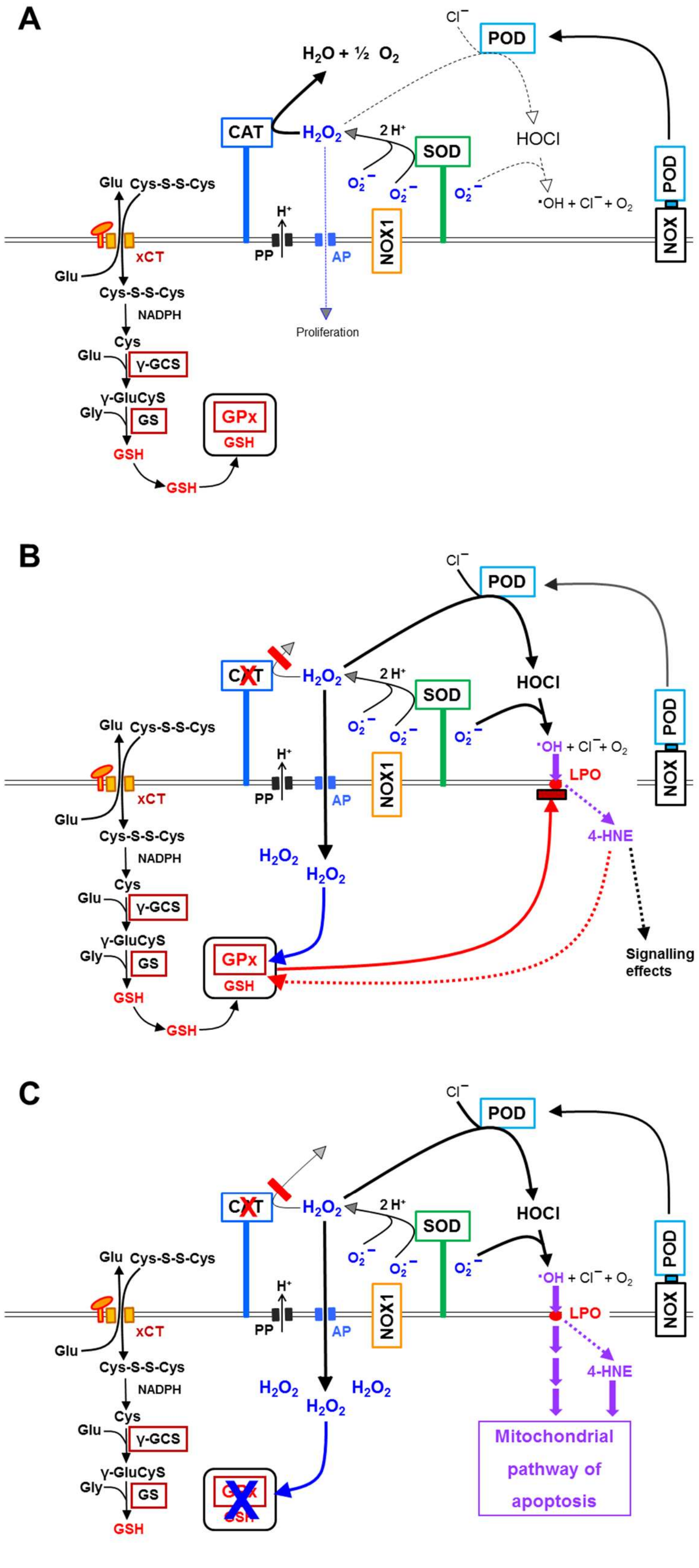

Supplementary Figure S7: **Redox biology at the membrane of bona fide tumor cells.** Please find details in the text.

However, the experimental data for tumor cells indicated that the effect based on NOX-1/catalase/aquaporins is dominant. This is explained by the generation of substantial concentrations of H_2O_2 through NOX-1 activity, followed by dismutation of superoxide anions, and by the high concentration of aquaporins in tumor cells [61].

The reflection on the analogous processes in transformed cells, i. cells from an early stage of oncogenesis (established by transformation *in vitro* without challenge by the natural tumor control systems that act *in vivo*) leads to the conclusion that their biochemical situation resembles that of tumor cells with inactivated catalase [13]. These processes are summarized in Supplementary Figure S8. The NOX-1-driven HOCl signaling pathway and the aquaporin-mediated influx of H_2O_2 derived from spontaneous dismutation of NOX-1-generated superoxide anions establish analogous effects on glutathione peroxidase/glutathione and apoptosis induction as seen before in the case of tumor cells with inhibited membrane-associated catalase. H_2O_2 thereby is the central player with a central role both in the establishment of HOCl signaling and in the depletion of glutathione, an essential step that is necessary to make HOCl signaling effective. For simplification, catalase is not shown in the scheme for transformed cells. In reality, transformed cells also express a low concentration catalase on their membrane [5]. However, in contrast to the locally high concentration of catalase on the membrane of tumor cells (that allows tight control of HOCl and NO/peroxynitrite signaling), the catalase concentration on transformed cells is too low to prevent intercellular ROS/RNS-dependent apoptosis-inducing signaling, It therefore only plays a modulatory role [5].

Supplementary Figure S8

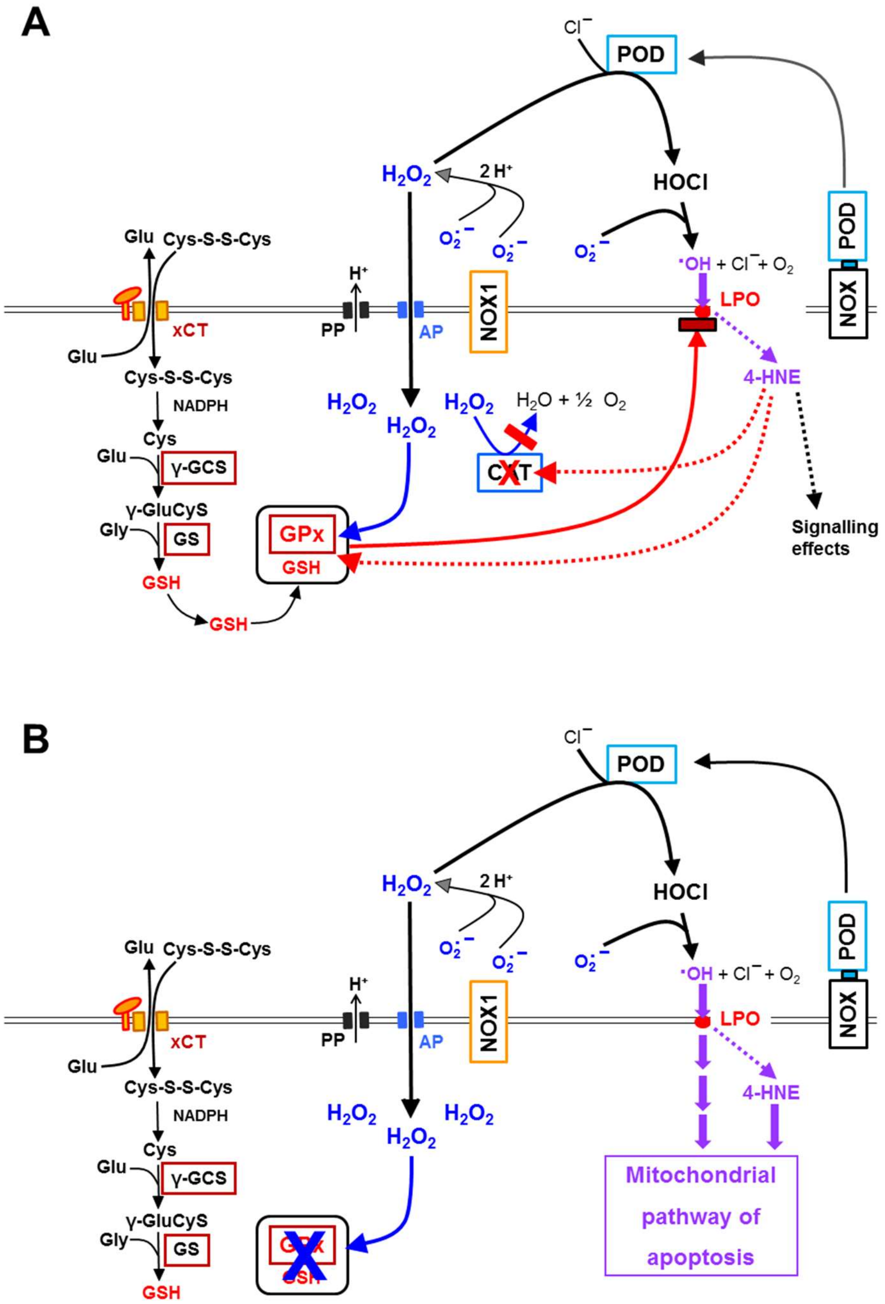

Supplementary Figure S8. Redox biology at the membrane of transformed cells, i. e. cells from early stages of oncogenesis or transformed *in vitro* without passage in animals.

As transformed cells only express minor concentrations of membrane-associated catalase (not shown in the figure), the biochemical processes are identical to the processes described for tumor cells with inhibited or inactivated catalase. NOX-1 expression is a hallmark of malignant transformation. H_2O_2 generated through dismutation of NOX-1 derived superoxide anions drives the HOCl signaling pathway and in parallel enters the cells through aquaporins. Glutathione depletion through H_2O_2 is necessary for abrogation of the inhibitory effect of glutathione peroxidase-

4/glutathione on lipid peroxidation-dependent apoptosis induction. 4-HNE might contribute to these processes which are, however, dominated by the effects of H_2O_2. Supplementary Figure S9

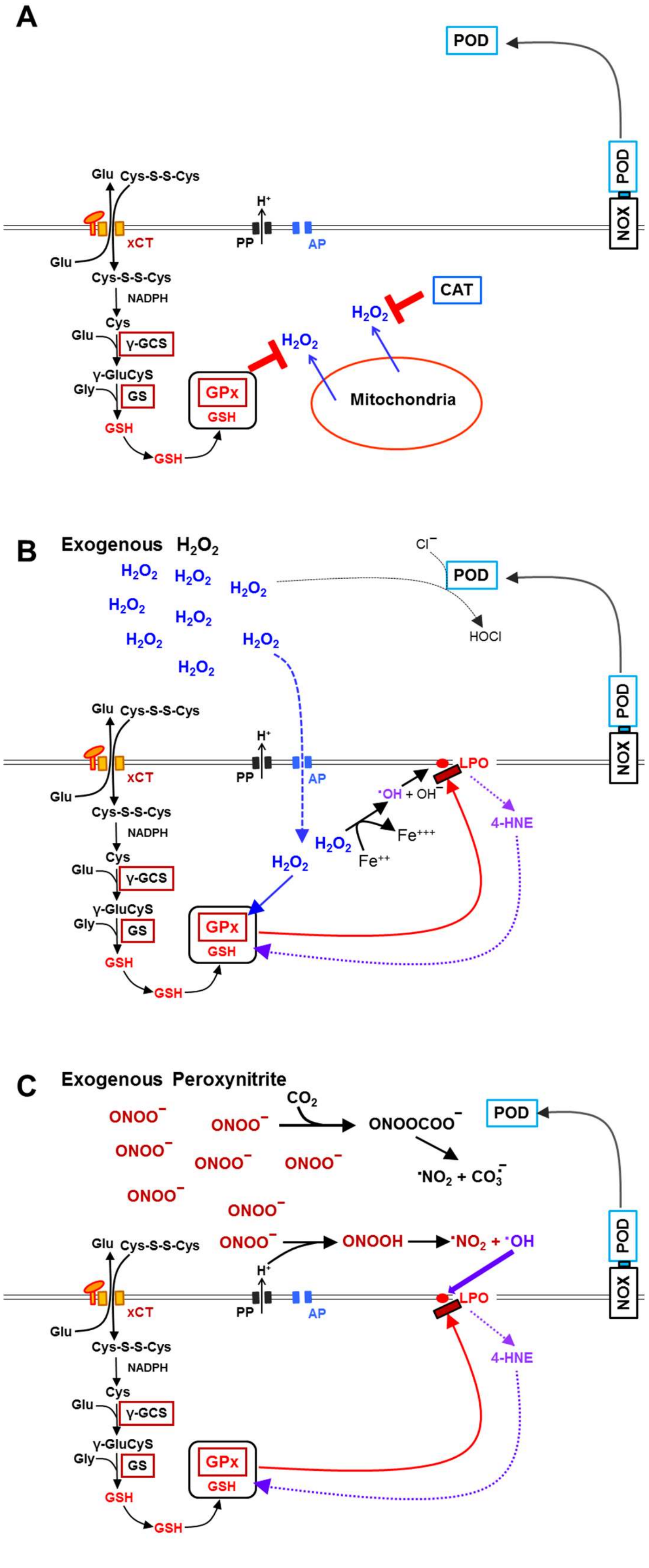

Supplementary Figure S9: Redox biology on the surface of non-malignant cells in the absence or presence of H_2O_2 or peroxynitrite. Please find details in the text. Nonmalignant cells neither express NOX-1 nor catalase on their membrane (Supplementary Figure S9 A). Due to missing extracellular superoxide anions, they cannot establish intercellular ROS/RNS signaling and do not respond to signaling components like peroxidase or NO. However, they can supplement malignant cells with extracellular peroxidase and thus enhance HOCl signaling of these malignant cells [27]. As non-malignant cells do not express membrane-associated catalase, they are not affected by the addition of catalase inhibitors or inactivators.

Even if the catalase inhibitor is cell-permeable, it does not have an effect on non-malignant cells, as long as their intracellular glutathione level is not reduced and therefore glutathione peroxidase/glutathione can take care of decomposition of H_2O_2 eventually generated by mitochondria.

Addition of exogenous H_2O_2 to non-malignant cells leads to H_2O_2 influx through aquaporins, followed by intracellular Fenton chemistry, resulting in lipid peroxidation through hydroxyl radicals [32] (Supplementary Figure S9 B). The apoptosis-inducing effect of lipid peroxidation is counterbalanced by glutathione peroxidase-4/glutathione, until glutathione depletion through H_2O_2 allows apoptosis induction by lipid peroxidation. Based on established findings, it is conceivable that 4-HNE contributes to glutathione depletion and to inhibition of glutathione peroxidase.

Addition of exogenous peroxynitrite to non-malignant cells leads to CO_2/peroxynitrite interaction, resulting in consumption of peroxynitrite and generation of NO_2 and carbonate radicals (Supplementary Figure S9C). In close vicinity to the proton pumps of the cells, protonation of peroxynitrite, resulting in the generation of peroxynitrous acid, allows for subsequent formation of hydroxyl radicals that cause lipid peroxidation. The activity of glutathione peroxidase-4/glutathione initially prevents lipid peroxide-dependent apoptosis induction. In this scenario, due to the absence of

H_2O_2, 4-HNE is the most likely candidate to inactivate glutathione peroxidase and to deplete glutathione. It thus allows the onset of apoptosis induction. This scenario is based on established findings but still requires experimental verification.

5. Supplementary References

1 Bauer G. Tumor cell protective catalase as a novel target for rational therapeutic approaches based on specific intercellular ROS signaling. Anticancer Res 2012; 32:2599-2624.

2 Bauer G. Targeting extracellular ROS signaling of tumor cells. Anticancer Res 2014; 34: 1467-1482.

3 Bauer G. Signal amplification by tumor cells: clue to the understanding of the antitumor effects of cold atmospheric plasma and plasma-activated medium. IEEE Transactions on Radiation and Plasma Medical Sciences 2018; 2:87-98.

4 Heinzelmann S, Bauer G. Multiple protective functions of catalase against intercellular apoptosis-inducing ROS signaling of human tumor cells. Biol Chem 2010; 391:675-693.

5 Böhm B, Heinzelmann S, Motz M, Bauer G. Extracellular localization of catalase is associated with the transformed state of malignant cells. Biol Chem 2015; 396:1339-1356.

6 Deichman GI, Vendrov EL. Characteristics of in vitro transformed cells essential for their in vivo survival, selection and metastatic activity. Int J Cancer 1986; *37*: 401-409.

7 Deichman GI, Kluchareva TE, Matveeva VA, Kushlinsky NE, Bassalyk LS, Vendrov EL. Clustering of discrete cell properties essential for tumorigenicity and metastasis. I. Studies of syrian hamster embryo fibroblasts spontaneously transformed in vitro. Int J Cancer 1989; *44*: 904-907.

8 Deichman G, Matveeva VA, Kashkina LM, Dyakova NA, Uvarova EN, Nikiforov MA, Gudkov AV. Cell transforming genes and tumor progression: *in vivo* unified secondary phenotypic cell changes. Int J Cancer 1998; *75*: 277-283.

9 Deichman G. Natural selection and early changes of phenotype of tumor cells *in vivo*: Acquisition of new defense mechanisms. Biochem 2000; *65*: 78-94.

10 Deichman G. Early phenotypic changes of *in vitro* transformed cells during *in vivo* progression: possible role of the host innate immunity. Sem Cancer Biol 2002; *12*: 317-326.

11 Irani K, Xia Y, Zweier JL, Sollott SJ, Der CJ, Fearon ER, Sundaresan M, Finkel T, Goldschmidt-Clermont PJ: Mitogenic signalling by oxidants in Ras-transformed fibroblasts. Science 1997; 275:1649-1652.

12 Irani K, Goldschmidt-Clermont PJ: Ras, Superoxide and Signal Transduction. Biochem Pharmacol 1998; 55:1339-1346.

13 Bauer G. HOCl and the control of oncogenesis. J Inorganic Biochem 2018; 179:10-23. doi:10.1016/j.jinorgbio.2017.11.005

14 Bauer G. Nitric oxide contributes to selective apoptosis induction in malignant cells through multiple reaction steps. Critical Reviews in Oncogenesis 2016; 21: 365-398, 2016

15 Herdener M, Heigold S, Saran M and Bauer G: Target cell-derived superoxide anions cause efficiency and selectivity of intercellular induction of apoptosis. Free Rad Biol Med 2000; *29*: 1260-1271.

16 Heigold S, Sers C, Bechtel W, Ivanovas B, Schäfer R, Bauer G. Nitric oxide mediates apoptosis induction selectively in transformed fibroblasts compared to nontransformed fibroblasts. Carcinogenesis 2002; 23: 929-941.

17 Bauer G. Increasing the endogenous NO level causes catalase inactivation and reactivation of intercellular apoptosis signaling specifically in tumor cells. Redox Biol 2015; 6: 353-371.

18 Bauer G. SiRNA-based analysis of the abrogation of the protective function of membrane-associated catalase of tumor cells. Anticancer Res 2017; 37: 567-582.

19 Bauer G and Motz M: The antitumor effect of single-domain antibodies directed towards membrane-associated catalase and superoxide dismutase. Anticancer Res 2016; 36: 5945-5956.

20 Bauer G, Sersenova D, Graves DB and Machala Z. Cold atmospheric plasma and plasma-activated medium trigger RONS- based tumor cell apoptosis. Sci Rep 2019; 9: 14210. https://doi.org/10.1038/s41598-019-50291-0

21 Bauer G, Sersenova D, Graves DB and Machala Z. Dynamics of singlet oxygen-triggered, RONS-based apoptosis induction after treatment of tumor cells with cold atmospheric plasma or plasma-activated medium. Sci Rep 2019; 9: 1393. https://doi.org/10.1038/s41598-019-50329-3

22 Yan DY, Talbot A, Nourmohammadi N, Sherman JH, Cheng XQ, Keidar M. Toward understanding the selective anticancer capacity of cold atmospheric plasma-A model based on aquaporins. Biointerphases 2015*;* 10: 040801.

23 Yan D, Xiao H, Zhu W, Nourmohammadi N, Zhang LG, Bian K, Keidar M. The role of aquaporins in the anti-glioblastoma capacity of the cold plasma-stimulated medium. J Phys D Appl Phys 2017; 50: 055401.

24 Bechtel W, Bauer G: Catalase protects tumor cells against apoptosis induction by intercellular ROS signaling. Anticancer Res 2009; 29: 4541-4557.

25 Bechtel W, Bauer G. Modulation of intercellular ROS signaling of human tumor cells. Anticancer Res 2009; 29: 4559-4570.

26 Engelmann I, Dormann S, Saran M, Bauer G: Transformed target cell-derived

superoxide anions drive apoptosis induction by myeloperoxidase. Redox Report 2000; 5: 207-214.

27 Pottgiesser S, Heinzelmann S and Bauer G. Intercellular HOCl-mediated apoptosis induction in malignant cells: interplay between NOX1-dependent superoxide anion generation and DUOX-related HOCl-generating peroxidase activity. Anticancer Res 2015; 35: 5927-5943.

28 Riethmüller M, Burger N, Bauer G: Singlet oxygen treatment of tumor cells triggers extracellular singlet oxygen generation, catalase inactivation and reactivation of intercellular apoptosis-inducing signaling. Redox Biol 2015; 6:157-168.

29 Bauer G. The synergistic effect between hydrogen peroxide and nitrite, two long-lived molecular species from cold atmospheric plasma, triggers tumor cells to induce their own cell death. Redox Biol 2019; 26: 101291. https://doi.org/10.1016/j.redox.2019.101291

30 Bienert GP, Schjoerring JK, Jahn TP. Membrane transport of hydrogen peroxide. Biochem Biophys Acta 2006; 1758: 994-1003.

31 Bienert GP, MØller AL, Kristiansen KA, Schulz A, MØller IM, Schjoerring JK, Jahn TP. Specific aquaporins facilitate the diffusion of hydrogen peroxide across membranes. J Biol Chem 2007; 282: 1183-1192.

32 Krüger H, Bauer G. Lactobacilli enhance reactive oxygen species-dependent apoptosis-inducing signalling. Redox Biol 2017; 11: 715-724.

33 Abdelrazzak AB, Stevens DL, Bauer G, O'Neill P and Hill MA (2011) The role of radiation quality in the stimulation of intercellular induction of apoptosis in transformed cells at very low doses. Rad. Res. 176, 346-355, 2011.

34 Bauer G. HOCl-dependent singlet oxygen and hydroxyl radical generation modulate and induce apoptosis of malignant cells. Anticancer Res 2013; 33: 3589-3602.

35 Saran M, Bors W: Signalling by O_2^- and NO: How far can either radical, or any specific reaction product, transmit a message under *in vivo* conditions? Chem Biol Interact 1994; 90: 35-45.

36 Denicola ABA, Freeman BA, Trujillo M, Radi R. Peroxynitrite reaction with carbon dioxide/bicarbonate: kinetics and influence on peroxynitrite-mediated reactions. Arch. Biochem. Biophys 1996; 333: 49-58.

37 Diatchuk V, Lotan O, Koshkin V, Wikstroem P and Pick E: Inhibition of NADPH oxidase activation by 4-(2-Aminoethyl-)benzenesulfonyl fluoride and related compounds. J Biol Chem 1997;272: 13292-13301.

38 Temme J, Bauer G. Low-dose gamma irradiation enhances superoxide anion production by nonirradiated cells through TGF-β1-dependent bystander signaling. Rad Res 2013;179: 422-432.

39 Bauer G, Bereswill S, Aichele P, Glocker E. Helicobacter pylori protects oncogenically transformed cells from reactive oxygen species-mediated intercellular induction of apoptosis. Carcinogenesis 2014; 35: 1582-1591.

40 Doctrow SR, Huffman K, Bucay Marcus C, Tocco G, Malfroy E, Adinolfi C, Kruk H, Baker K, Lazarowych N, Mascarenhas J and Malfroy B: Salen-manganese complexes as catalytic scavengers of hydrogen peroxide and cytoprotective agents: structure-activity relationship studies. J Med Chem 2002; 45: 4549-4558.

41 Abashkin YG, Burt SK.(Salen)MnIII compounds as nonpeptidyl mimics of catalase. Mechanism-based tuning of catalase activity: a theoretical study. Inorg Chem 2005; 44: 1425-1432.

42 Ophoven S, Bauer G. Salen manganese complexes: sophisticated tools for the analysis of intercellular ROS signaling pathways. Anticancer Res 2010; 30: 3967-3980.

43 Bauer G. Central signaling elements of intercellular reactive oxygen/nitrogen species-dependent induction of apoptosis in malignant cells. Anticancer Res 2017; 37:499-514.

44 Kettle AJ, Gedye CA, Hampton MB, Winterbourn CC: Inhibition of myeloperoxidase by benzoic acid hydrazides. Biochem J 1995;308: 559-563.

45 Kettle AJ, Gedye CA, Winterbourn CC: Mechanisms of inactivation of myeloperoxidase by 4-aminobenzoic acid hydrazide. Biochem J 1997; 321: 503-508.

46 Burner U, Obinger C, Paumann M, Furtmüller PG, Kettle AJ. Transient and steady-state kinetics of the oxidation of substituted benzoic acid hydrazides by myeloperoxidase. J Biol Chem 1999; 274: 9494-9502.

47 Aruoma OI, Halliwell B, Hoey BM, Butler J: The antioxidant action of taurine, hypotaurine and their metabolic precursors. Biochem J 1988; 256: 251-256.

48 Goldstein S, Czapski G. Mannitol as an $OH^{\cdot}$ scavenger in aqueous solutions and in biological systems. Int J Rad Biol 1984;46: 725-729.

49 Steinebach C, Bauer G. An alternative signalling pathway based on nitryl chloride during intercellular induction of apoptosis. In vitro and applied molecular toxicology 2001;14: 107-120.

50 Pfeiffer S, Leopold E, Schmidt K, Brunner F, Mayer B. Inhibition of nitric oxide synthesis by NG-nitro-L-arginine methyl ester (L-NAME): requirement for

bioactivation to the free acid, NG-nitro-L-arginine. Br J Pharmacol 1996;118:1433-1440.

51 Salvemini D, Wang Z Q, Stern MK, Currie M G, Misko TP. Peroxynitrite decomposition catalysts: therapeutics for peroxynitrite-mediated pathology. Proc Natl Acad Sci USA 1998;95: 2659–2663.

52 Jensen MP, Riley DP. Peroxynitrite decomposition activity of iron porphyrin complexes. Inorg Chem 2002; 41: 4788–4797.

53 Gout PW, Buckley AR, Simms CR, Bruchovsky N. Sulfasalazine, a potent suppressor of lymphoma growth by inhibition of the x(c)- cystine transporter: a new action for an old drug. Leukemia 2001; 15:1633–1640.

54 Zucker B, Hanusch J, Bauer G. Glutathione depletion in fibroblasts is the basis for induction of apoptosis by endogenous reactive oxygen species. Cell Death and Differentiation 1997; 4: 388-395.

55 Putnam CD, Arvai AS and Bourne Y: Active and inhibited human catalase structures: Ligand and NADPH binding and catalytic mechanism. J Mol Biol 2000; 296: 295-309.

56 Scheit K, Bauer G. Direct and indirect inactivation of tumor cell protective catalase by salicylic acid and anthocyanidins reactivates intercellular ROS signaling and allows for synergistic effects. Carcinogenesis 2015; 36; 400-411.

57 Kerr, JFR, Wyllie AH, Currie AR. Apoptosis: a basic biological phenomenon with wide-ranging implications in tissue kinetics. Br J Cancer 1972; 26: 239-257.

58 Elmore S. Apoptosis: A review of programmed cell death. Toxicol Pathol 2007; *35*: 495-515.

59 Hipp M-L, Bauer G. Intercellular induction of apoptosis in transformed cells does not depend on p53. Oncogene 1997;15: 791-797.

60 Beck E, Schäfer R, Bauer G. Sensitivity of transformed fibroblasts for intercellular induction of apoptosis is determined by their transformed phenotype. Exp Cell Res 1997; 234: 47-56.

61 Verkman AS, M. Hara-Chikuma M, Papadopoulos MC. Aquaporins – new players in cancer biology. J Mol Med 2008; 86:523-529.
